# Supplementary material for: Preventing childhood obesity, phase II feasibility study focusing on South Asians: BEACHeS
Source: BMJ Open. 2014 Apr 10;4(4):e004579. doi: 10.1136/bmjopen-2013-004579 (PMC3987740; doi:10.1136/bmjopen-2013-004579)
Supplement: Author's manuscript [file bmjopen-2013-004579.draft_revisions.pdf]

Preventing childhood obesity, exploratory trial focusing on South Asians: BEACHeS

Journal:	*BMJ Open*
Manuscript ID:	bmjopen-2013-004579
Article Type:	Research
Date Submitted by the Author:	29-Nov-2013
Complete List of Authors:	Adab, Peymane; The University of Birmingham, Public Health, Epidemiology & Biostatistics<br>Pallan, Miranda; University of Birmingham, Public Health, Epidemiology & Biostatistics<br>Cade, Janet; University of Leeds,<br>Ekelund, Ulf; MRC Epidemiology Unit,<br>Barrett, Timothy; Birmingham Childrens HospitalUniversity of Birmingham, Diabetes Unit Institute of Child Health<br>Daley, Amanda; University of Birmngham, Primary Care Clinical Sciences<br>Deeks, Jon; University of Birmingham, Public Health, Epidemiology & Biostatistics<br>Duda, Joan; University of Birmingham, Sport, Exercise & Rehabilitation Sciences<br>Gill, Paramjit; University of Birmingham, Primary Care Clinical Sciences<br>Parry, Jayne; University of Birmingham, Public Health, Epidemiology & Biostatistics<br>Bhopal, Raj; University of Edinburgh<br>Cheng, Kar Keung; The University of Birmingham, Public Health, Epidemiology & Biostatistics
**Primary Subject Heading**:	Public health
Secondary Subject Heading:	Public health
Keywords:	Obesity, Children, Prevention

SCHOLARONE™
Manuscripts

Title: Preventing childhood obesity, exploratory trial focusing on South Asians: BEACHeS

Authors: Peymané Adab (MD)[1], Miranda Pallan (PhD)[1], Janet Cade (PhD)[2], Ulf Ekelund (PhD)[3], Timothy Barrett (PhD)[4], Amanda Daley (PhD)[1], Jonathan Deeks (PhD)[1], Joan Duda (PhD)[5], Paramjit Gill (DM)[1], Jayne Parry (MD)[1], Raj Bhopal (PhD)[6], KK Cheng (PhD)[1]

Affiliations:
[1]School of Health & Population Sciences (Public Health, Epidemiology and Biostatistics and Primary Care Clinical Sciences), University of Birmingham
[2]Nutritional Epidemiology Group, School of Food Science and Nutrition, University of Leeds
[3]MRC Epidemiology Unit, Institute of Metabolic Science, Cambridge & Department of Sport Medicine, Norwegian School of Sport Sciences
[4]School of Clinical and Experimental Medicine, University of Birmingham
[5]Sport & Exercise Sciences, The University of Birmingham
[6]Edinburgh Ethnicity and Health Research Group, Centre for Population Health Sciences, University of Edinburgh

Address correspondence to:
Miranda J Pallan, Public Health, Epidemiology and Biostatistics, University of Birmingham, Edgbaston, Birmingham, B15 2TT, UK.
Tel: +441214147990
Fax: +441214147878
E-mail: m.j.pallan@bham.ac.uk

Key words: Childhood obesity prevention; exploratory trial; MRC framework for complex interventions

Word count:
Abstract: 273
Manuscript: 3366

For peer review only

Abstract

Objectives: To assess feasibility and acceptability of a multifaceted, culturally appropriate intervention for preventing obesity in South Asian children, and to obtain data to inform sample size for a definitive trial.

Design: Phase II exploratory trial of a complex intervention.

Setting: Eight primary schools in inner city Birmingham, UK, within populations that are predominantly South Asian.

Participants: 1090 children aged 6-8 years took part in the intervention. 571 (85.9% from South Asian background) underwent baseline measures. 85.5% (n=488) were followed up 2 years later.

Interventions: The one-year intervention consisted of school and family based activities, targeting dietary and physical activity behaviours. The intervention was modified and refined throughout the period of delivery.

Main outcome measures: Acceptability and feasibility of the intervention and of measurements required to assess outcomes in a definitive trial. The difference in BMI z-score between arms was used to inform sample size calculations for a definitive trial.

Results: Some intervention components (increasing school physical activity opportunities, family cooking skills workshops, signposting of local leisure facilities and attending day event at a football club) were feasible and acceptable. Other components were acceptable, but not feasible. Promoting walking groups was neither acceptable nor feasible. At follow up, children in the intervention compared with the control group were less likely to be obese (OR 0.41; 0.19 to 0.89), and had lower adjusted BMI z-score [-0.15 (-0.27, -0.03)].

Conclusions: The exploratory trial informed components for an intervention programme. The favourable direction of outcome for weight status in the intervention group supports the need for a definitive trial. A cluster randomised controlled trial is now underway to assess the clinical and cost-effectiveness of the intervention.

For peer review only

Article Summary

Strengths and limitations of the study

- We report the findings of an exploratory trial of a childhood obesity prevention intervention that focuses on primary school-aged children from South Asian communities in the UK. Despite their susceptibility to the cardiometabolic consequences of obesity, little obesity prevention research has been undertaken in these communities previously.
- The early phases of the UK Medical Research Framework for complex health interventions has guided the intervention development and evaluation process undertaken in this exploratory trial.
- The feasibility and acceptability of the childhood obesity prevention intervention components was variable and context dependent, however, the exploratory nature of the trial enabled us to modify and refine delivery of the intervention throughout.
- Development and evaluation of the feasibility and acceptability of the intervention was undertaken in materially disadvantaged, predominantly South Asian communities, thus its transferability would be dependent on tailoring to the specific local context.
- The final intervention programme, following modification and refinement in this exploratory trial, is being definitively evaluated in an ongoing cluster-randomised controlled trial.

For peer review only

Introduction

Childhood obesity is a growing problem worldwide.[1] Apart from psychological and social problems, longitudinal studies show adverse future health consequences in children as young as 7 years old who are obese.[2] In the UK, although childhood overweight prevalence has stabilised, socioeconomic disparities have widened, with increasing trend in more deprived sub-populations.[3] Data from the national childhood surveillance programmes in England show that at school entry (age 4-5 years), 9.5% of children are obese (i.e., above 95th percentile for national reference standards), but this prevalence doubles (19.2%) by the end of primary school (age 11).[4] The rate of increase among children from South Asian (SA) ethnic groups, especially girls, is greater than that for the population as a whole (increasing trend of 1.13% and 0.66% per year for Bangladeshi and Pakistani girls respectively, compared with 0.35% yearly increase in White British).[5] Thus the primary school period presents a key phase for prevention, and SA are an important target group.

However, despite numerous systematic reviews,[6,7] reports[8,9] and guidelines,[10] evidence for effective approaches to prevention is limited, particularly among minority ethnic groups. Relevant trials suggest that multifaceted school-based interventions have potential, particularly those that also include a home or community element, but the most effective combination of components is not clear.[7,9] The need for involving stakeholders, such as families, schools and local communities, in the decision making regarding potential intervention strategies has been highlighted.[6] Furthermore, for a complex intervention such as obesity prevention, which has several interconnecting components, a rigorous and iterative phased approach is required to improve study design, execution and applicability of results. The UK Medical Research Council (MRC) proposed a framework for such interventions.[11] Given the growing problem of obesity and lack of clarity on effective approaches to

For peer review only

prevention, it would be unwise to embark on another trial without thorough attention to the early phases described in the MRC framework.

The Birmingham healthy Eating and Active lifestyle for CHildren Study (BEACHeS http://www.birmingham.ac.uk/research/activity/mds/projects/HaPS/PHEB/WAVES/BEACHeS/index.aspx), used the theoretical and modelling phases of the MRC framework to develop a multifaceted childhood obesity prevention programme, targeting SA children (phase I).[12] Here we report on the exploratory trial (phase II). The aim was to assess feasibility and acceptability of the intervention. In addition we wanted to obtain data to inform a definitive (phase III) cluster-randomised controlled trial (RCT).

For peer review only

Materials and Methods

The exploratory trial was conducted in eight Birmingham primary schools from 2006 to 2009. Children underwent baseline measures between December 2006 and June 2007. Four schools were selected to receive the intervention (2007/8 academic year), and the remainder had no active intervention. Follow up data were collected two years after baseline.

Setting

Birmingham is UK's second city with a high minority ethnic population (34%), one fifth being from the three main SA communities (Pakistani, Bangladeshi and Indian). We obtained a list of all Local Authority-maintained primary schools in Birmingham. Of 304 schools, 52 had ≥50% of pupils from SA background (mean 75%). These, compared with the remainder, had a higher proportion of children eligible for free school meals (FSM), indicating higher deprivation. Schools were ranked in order of FSM eligibility, and those from either extreme were successively invited until 8 agreed to take part.

Participants

Pupils from years 1 and 2 (aged 5-7 years) were invited to participate. Parents of the children were approached by letter distributed through the schools, and active consent was sought for their child to participate in measurements.

Baseline and follow up measures

Age, sex and ethnicity data (from parent report at school entry) were obtained from school records on all eligible children in participating schools. Children with consent also underwent a range of anthropometric measurements, including standing height (measured to nearest 0.1cm with a Leicester Height Measure), weight (measured to nearest 0.1 kg with aTanita bioimpedance monitor), two measures of waist circumference (measured to nearest 0.5cm),

For peer review only

and skinfold thickness at five sites (biceps, triceps, subscapular, suprailiac and thigh; measured using a Holtain calliper). Children also completed interviewer administered questionnaires (not discussed in this paper, but including: quality of life (PedsQL),[13] self concept (Marsh self-description questionnaire),[14] perceived physical competence (Harter Pictorial Scale for Young Children),[15] and body image perception (adapted Collin's Pictorial Image Scale)[16]). All measures were undertaken by trained researchers using standard protocols.

Dietary intake was assessed using the Child And Dietary Evaluation Tool (CADET)[17]; a 24-hour food tick list that has been validated against a semi-weighed diary in children aged 3-7 years. A researcher completed the CADET for children during school hours, and parents were given instructions for completing it for the remainder of the 24 hour period. Physical activity levels were assessed using the Actiheart monitor (CamnTech, Papworth UK) worn for five consecutive days, including a weekend. This is validated for use in children[18] and was set up to measure acceleration and heart rate at 30 second epochs. In addition, parents were asked to complete questionnaires which included questions on family composition, and family dietary and physical activity habits.

Intervention

The process for intervention development has been reported elsewhere,[12] but in brief, the multicomponent intervention was developed by combining evidence from the literature with views from key stakeholders (including parents, teachers, school nurses, dieticians, community leaders, school governors, and retail and leisure representatives close to schools) and a multi-disciplinary group of relevant professionals. Important contextual data were gained from stakeholders, which was critical for informing intervention development.[19] A review of local facilities, resources and opportunities related to healthy eating and the promotion of physical activity targeting children was used to inform the design and

For peer review only

encourage longer term sustainability of the intervention. We also took account of national childhood obesity prevention policy during the development process to try and ensure that the intervention had an impact that was additional to existing national initiatives. The intervention targeted both diet and physical activity behaviours and consisted of two main strands: i) increasing children's physical activity levels and promoting healthy eating through schools, and ii) increasing skills among family members through family educational activities. A number of intervention techniques (as defined in the CALO-RE Taxonomy of behaviour changed techniques for physical activity and healthy eating[20]) were utilised to deliver each intervention component. A more detailed description of the intervention is provided in Table 1.

For peer review only

Table 1: Intervention components and techniques[20] included in the BEACHeS intervention programme and findings from the process evaluation

Intervention component	Aim	Intervention techniques	Description	Evaluation method	Evaluation findings
			**School based activities**		
Physical activities within school day	To increase the amount of time that children are physically active within the school day	Environmental restructuring<br>Prompt practice	Three elements introduced into schools:<br>1. 'Wake Up Shake Up': a short (10 minutes) organised daily dance or exercise routine to music<br>2. Organised playground activities at lunch and break times through the training of school staff to act as "play leaders"<br>3. 'Take 10': teaching resource which links 10 minutes physical activity in the classroom to curricular subjects.	• Interviews with school staff<br>• Observation of sessions in schools<br>• Self-completion questionnaires administered to children and parents	Overall, school staff with a responsibility for health were enthusiastic and committed to introducing these schemes, and all schemes were acceptable to children. Individual school and staff factors strongly influence the success of each element in the different schools. Parents, in general felt that the amount of physical activity their children were undertaking in school had increased over the last year.
Incentive scheme to encourage physical activity out of school	To increase the amount of time outside of school hours that children spend doing leisure physical activities	Prompt self monitoring of behaviour<br>Prompt practice<br>Provide rewards contingent on successful behaviour	Children received a sticker collection card from school and information on local participating sports and leisure venues. Each time a child attended a venue, they collected a sticker. The child with the most stickers in each school received a prize.	• Interviews with school staff<br>• Telephone survey of leisure venue staff<br>• Assessment of returned collection cards<br>• Questionnaires to children	Although this type of incentive scheme appears acceptable to children, parents and school staff alike, it was not feasible in terms of maintaining cooperation of participating venues. An element that was well received and could be retained, is the signposting information given to children and families.
Attendance at a course run by a Premier league football club	To encourage physical activity and healthy eating through an iconic sporting institution	Provide information on consequences of behaviour<br>Model/demonstrate behaviour<br>Prompt identification of role model/advocate<br>Goal setting	School classes attend a 'Villa Vitality' day. Half the day is spent with Football Club coaches, exercising and learning football skills, and the other half of the day is an interactive learning session on healthy eating and healthy lifestyles. Teachers provided with material to deliver over 6 weeks to reinforce messages, and	• Interviews with school staff<br>• Self-completion questionnaires to parents<br>• Pre- and 6-week post intervention questionnaires (knowledge, attitudes & behaviour) administered	This was highly acceptable to children and school staff and is feasible to deliver to the target age group. There is some evidence that it may favourably alter children's health-behaviours.

For peer review only

		(behaviour)	encourage weekly challenges or goals.	to children	
			**Increasing skills of families through activity-based learning**		
Cooking courses for family members	To increase healthy cooking skills and confidence and influence dietary behaviour	Provide information on consequences of behaviour in general<br>Model/demonstrate behaviour<br>Provide instruction on how to perform the behaviour<br>Prompt generalisation of behaviour	Five week courses on healthy cooking were delivered through schools to parents or other family members, some courses include children. Courses ran successively to allow all parents to attend if they wanted. Healthy recipes were distributed to support the course content.	• Interviews with school staff<br>• Uptake rates for courses<br>• Participant pre and post-course questionnaires	This component was popular with those who participated and there was some evidence that it influenced confidence and cooking practices. Running sessions for parents and children was the most popular model, and having the sessions based in school time for children and inviting parents to attend improved attendance.
Information on local leisure opportunities and "taster" sessions for families	To equip families with the knowledge and skills to undertake physical activities with their children in their leisure time	Provide information on when and where to perform behaviour<br>Model/demonstrate behaviour	Parents were given information on local sporting and leisure venues and events. They were invited to attend weekend taster sessions with their children, through school. Activities range from cricket and football, to archery, climbing and dry-slope skiing. There was no cost for the activities and transport was provided.	• Interviews with school staff<br>• Uptake of the taster sessions<br>• Self-completion questionnaires to parents and children	This component was resource intensive to deliver, and uptake was very low. However, the signposting information was used by families, and was appreciated.
Training walk leaders to initiate community walking programmes	To increase walking by families and other community members through organised leisure walks	Model/demonstrate behaviour<br>Prompt practice	Community volunteers were recruited through schools to become trained walk leaders. Training was provided to equip volunteers to organise and lead walks in their local community.	• Assessment of numbers attending training to be walk leaders<br>• Monitoring numbers joining walking groups	This component proved unfeasible, as there was a lack of volunteers. Even those who expressed an initial interest failed to attend the training. Despite repeated efforts to recruit community volunteers, no one attended the training in any of the four BEACHeS intervention communities.

For peer review only

Allocation of intervention

This was a non-randomised feasibility trial. Intervention allocation was by school, matching pairs of schools by size, geographical location and proportion of children eligible for FSM. The pairs were then randomly allocated to intervention or control arm.

Process measures

The main aim of the study was to assess intervention feasibility and acceptability. Each component was evaluated separately, using a variety of methods. These included collection of uptake data, direct observation, questionnaires to children and parents and interviews with key school staff. The questionnaires were also used to evaluate overall perceptions of the intervention and engagement with different intervention components. Topics covered in the semi-structured interviews included exploration of how the different intervention components were implemented, which elements were perceived to work well and ideas for further development. The interviews were tape-recorded, transcribed and analysed thematically.

Other measures and analysis

We assessed the feasibility of obtaining outcome data, primarily body mass index (BMI), but also diet and physical activity and other anthropometric measures as described above. Exploratory comparison between intervention and control children was also undertaken to determine effect size.

Height and weight data were used to calculate BMI (kg/m^2) and converted into standard deviation scores (BMI z-score) using the UK 1990 growth reference charts.[21] Children were categorised as underweight, healthy weight, overweight or obese using the 2nd, 85th and 95th centile cut-offs. For waist circumference and skinfolds, the mean was used for analyses. Skinfold measures were combined to obtain sum, upper (biceps, triceps and subscapular) and lower (suprailiac and thigh) skinfolds.

For peer review only

Data from the CADET were coded and analysed by a food diary analysis program (DANTE, University of Leeds) to estimate total energy intake (Kcal) and amount of fruit and vegetables, and sugar consumed. Data on foods consumed in school and at home were analysed separately, then combined to obtain estimates for the complete 24 hour measurement period.

Accelerometry data were used to assess physical activity levels (http://www.mrc-epid.cam.ac.uk/Research/Programmes/Programme_5/InDepth/Programme%205_Downloads.html). Total daily volume of physical activity was estimated and expressed as average counts per minute (cpm). The mean duration of daily moderate to vigorous physical activity (MVPA, min/day) was calculated (400 cpm cut-off for lower threshold).[22] The proportion participating in ≥60 minutes MVPA (as recommended by international guidelines) was also calculated.

The intraclass correlation coefficient (ICC) for the main outcome (BMI z-score) was calculated, but because of the small number of schools, clustering was not taken into account for the analysis. We analysed final BMI, diet and physical activity levels of children in the intervention, compared with those in the control group, adjusted for baseline measures. We used multivariate analysis to adjust for potential confounders (age, sex, ethnicity and baseline values). Logistic regression was used to assess risk of obesity, and likelihood of meeting ≥60 minutes MVPA at follow up in the intervention, compared with control children.

Ethical approval

Ethical approval was granted by The Black Country Research Ethics Committee (08/H1202/22). Approval was sought for active consent from parents for the child measurements, whilst consent for participation in the intervention was at school level.

For peer review only

Results

Feasibility and acceptability of intervention components

Some intervention components (particularly those delivered through school) were more successfully delivered than others. Intervention was modified during the course of delivery to optimise participation and in response to feedback. The findings are summarised in Table 1, and details are reported in the appendix.

Two intervention components were found to be unsuitable to include within an intervention programme in the format delivered. First was the scheme to incentivise out of school leisure activities. Poor co-operation from leisure venues and lack of resources to continually remind and motivate children contributed to the failure of this component. Second was the training of walk leaders. Despite effort to recruit through school staff, influential parents and various forms of publicity, volunteers were not forthcoming. The only person who underwent training did not undertake any walking groups.

One component was partially successful. Signposting of leisure facilities in the local area was popular among parents and school staff. However, attendance at organised taster sessions was poor, which was outweighed by the high staff and monetary resources required to deliver the component. The taster sessions were therefore not feasible to include in a larger trial.

The other intervention components had varying degrees of success, and the process evaluation highlighted how delivery could be improved. Individual school characteristics and differences between staff members strongly influenced the success of each element in the different schools.

For peer review only

Acceptability of allocation to control

Acceptability of non-intervention was assessed through interviews with control school staff. All understood the need for a control arm. One would have liked alternative support to compensate for not being offered the intervention. In other control schools, staff expressed that being part of the study had benefitted them in other ways, and contributed to the school's status as a 'healthy school'.

Outcomes

Feasibility of outcome measures

There were 1090 eligible children in the eight participating schools (range 54-180). Of these, 606 (55.6%) had parental consent and anthropometric measures were completed on those in school on measurement days (n=571, 94.2% of consented). Useable data (≥3 days) for Actiheart were available for 508 (89.0%). Completion of CADET was more variable. Although 445 (77.9%) were returned at baseline, 269 (47.1%) were complete, of which two thirds (n=174) had usable data. Two years after the baseline measures, 488 children (85.5%) were successfully followed up. The proportion with usable Actiheart data was similar to baseline. However a higher proportion (n=454, 93%) had a completed CADET, although only 163 (36%) had usable home data.

Findings from exploratory trial

A total of 574 children were included in the trial (Figure 1), of whom 85.9% were SA. Baseline characteristics are summarised in Table 2 (anthropometric measures were completed for 571 of the 574 participating children). The age, sex and ethnicity of those who took part were similar in distribution to the characteristics of the non-consented eligible children.

For peer review only

Over 90% in both arms were from the most deprived areas, in keeping with the location and ethnic composition of the population. Around one in five (n=115) were overweight or obese. This proportion was slightly higher in the control (21.7%) compared to the intervention (18.3%) schools, mainly due to a higher prevalence of overweight. A similar pattern was seen for other measures of body fat (skinfold measures, bioimpedance), but not for waist circumference which was similar in intervention and control groups. Under half of the children (47.9%) undertook ≥60 minutes of MVPA. Levels of physical activity (total cpm) and duration of time spent in MVPA were slightly higher among children from control, compared with intervention schools. Total dietary energy, fruit and vegetable and sugar intake were slightly higher among children in intervention, compared with control schools.

Two-years post baseline, 254 (83.3%) children in the control and 234 (86.2%) in the intervention schools were successfully followed up. There was no significant difference in baseline weight status, MVPA, diet or sex, between those followed up, and those lost to follow up (data not shown). However, SA children were less likely to be lost to follow up (n=58; 11.9%) compared with those from other ethnic groups (n=28; 34.6%).

For peer review only

Table 2: Baseline characteristics of children measured for the BEACHeS exploratory trial

	Intervention group (N=269)	Control group (N=305)
Sex : Number (%)		
Male	144 (53.5)	142 (49.8)
Female	125 (46.5)	153 (50.2)
Mean age in years (SD)	6.53 (0.59)	6.44 (0.58)
Ethnicity: Number (%)		
Bangladeshi	36 (13.4)	46 (15.1)
Indian	22 (8.2)	5 (1.6)
Pakistani	181 (67.3)	203 (66.6)
Other	30 (11.2)	51 (16.7)
Townsend score decile: Number (%)		
1 (most deprived)	250 (93.3)	285 (93.8)
2	6 (2.2)	9 (3.0)
3	8 (3.0)	5 (1.6)
Mean BMI-SDS score (SD)	-0.03 (1.37)	0.08 (1.39)
Weight status: Number (%)		
Underweight	6 (2.3)	10 (3.3)
Healthy weight	212 (79.4)	228 (75.0)
Overweight	15 (5.6)	27 (8.9)
Obese	34 (12.7)	39 (12.8)
Mean waist circumference in cm (SD)	55.6 (7.7)	55.3 (6.9)
Skinfold measures in mm: mean (SD)		
Biceps	7.5 (3.6)	8.0 (4.0)
Triceps	10.9 (4.1)	11.6 (4.5)
Subscapular	7.5 (4.4)	7.9 (5.0)
Suprailiac	7.0 (4.4)	7.4 (4.7)
Sum of 4 skinfolds	32.5 (14.7)	34.8 (16.8)
Thigh	14.4 (5.5)	15.7 (6.3)
Sum of upper skinfolds	25.7 (10.9)	27.5 (12.5)
Sum of lower skinfolds	21.0 (8.8)	22.9 (10.1)
Mean (SD) bioimpedance (Ω)	692.6 (72.5)	695.1 (80.8)
Physical activity (SD)		
Mean counts/min	79.9 (23.4)	83.4 (27.3)
Mean MVPA min/24h	52.8 (28.4)	62.9 (25.0)
Number (%) achieving ≥60 mins MVPA	100 (40.2)	156 (54.5)
1 day school dietary intake (SD)		
Mean energy (Kcal)	568.4 (387.0)	458.2 (435.4)
Fruit and vegetables (grams)	140.6 (121.4)	105.8 (118.7)
Sugar (grams)	41.0 (60.9)	35.3 (62.9)
1 day home dietary intake (SD)		
Mean energy (Kcal)	1678.12 (760.30)	1555.53 (750.04)
Fruit and vegetables (grams)	329.85 (232.23)	267.95 (193.27)
Sugar (grams)	105.36 (50.20)	93.50 (36.63)
24h dietary intake: Mean (SD)		
Total energy (Kcal)	2229.1 (909.1)	2007.0 (964.5)
Fruit and vegetables (grams)	475.6 (261.4)	368.7 (220.2)
Sugar (grams)	154.1 (108.7)	129.7 (85.8)

For peer review only

Estimation of effect size to inform definitive trial

At follow up, the proportion of children who were overweight or obese had increased in all schools (from 7.3% to 9.9%, and from 12.8% to 19.1% for overweight and obese respectively). The risk of obesity was significantly lower in the intervention compared with the control group (OR 0.41; 0.19 to 0.89). The increase in BMI z-score was also significantly lower in the intervention compared with control, after adjustment [-0.15kg/m^2 (-0.27, -0.03)] (Table 3). A similar trend was seen for all other anthropometric measures, although none were statistically significant.

The ICC for the outcome "overweight/obese" compared to non-overweight, was 0.00 (95% CI (0, 0.02), whilst for BMI z-score, the ICC was 0.01 (95% CI (0, 0.04)). Therefore, taking account of clustering in the analysis would make marginal difference to the findings.

The proportion of children who undertook ≥60 minutes MVPA reduced (from 48.8% to 27.1%) at follow up, with the reduction being greater among control (30.2%) compared with intervention (23.8%) children. The differences in physical activity levels at follow up were not significant between groups (Table 3).

Total calorific intake had increased slightly at follow up (1786 Kcal at baseline to 1943 Kcal at follow up). There were no significant differences in dietary intake between control and intervention children, although 24 hour dietary intake data were only available for 33%. However, school dietary intake data were more complete (93%), and children in intervention schools had significantly more fruits and vegetables and lower sugar intake, compared with those in control schools (Table 3).

For peer review only

Table 3: Anthropometric, diet and physical activity measures at follow up, and adjusted difference in measures between control and intervention groups

	Intervention group (N=234)	Control group (N=254)	Intervention vs. Control (adjusted for baseline)	p value	Intervention vs. Control (finally adjusted)*	p value
Number (%)			***OR (95% CI)***		***Adjusted* OR (95% CI)***	
Obese	36 (15.4)	57 (22.4)	0.36 (0.17, 0.77)	0.01	0.41 (0.19, 0.89)	0.02
Achieving ≥60 mins MVPA	53 (23.6)	73 (30.2)	0.82 (0.52, 1.28)	0.38	0.74 (0.45, 1.20)	0.22
Mean (SD)			***B (95%CI)***		***Adjusted* B (95%CI)***	
**Anthropometric measures**						
BMI z-score	0.13 (1.5)	0.40 (1.5)	-0.15 (-0.26, -0.03)	0.02	-0.15 (-0.27, -0.03)	0.02
Waist circumference (cm)	59.4 (9.5)	60.4 (9.1)	-0.88 (-1.87, 0.10)	0.08	-0.86 (-1.87, 0.15)	0.09
Skinfold measures (mm)						
Biceps	6.9(3.5)	7.7 (3.8)	-0.48 (-0.98, 0.01)	0.06	-0.44 (-0.93, 0.06)	0.08
Triceps	11.2 (4.8)	11.9 (4.6)	-0.14 (-0.68, 0.40)	0.61	-0.10 (-0.64, 0.45)	0.71
Subscapular	8.5 (5.3)	9.3 (5.8)	-0.46 (-0.98, 0.06)	0.09	-0.38 (-0.89, 0.14)	0.15
Suprailiac	8.8 (5.9)	9.4 (5.9)	-0.23 (-0.84, 0.37)	0.45	-0.23 (-0.83, 0.37)	0.46
Sum of 4 skinfolds	35.2 (18.1)	37.6 (18.4)	-1.09 (-2.85, 0.67)	0.23	-0.97 (-2.70, 0.77)	0.27
Thigh	17.3 (7.5)	18.9 (8.1)	-0.31 (-1.39, 0.78)	0.58	-0.27 (-1.38, 0.84)	0.63
Sum of upper skinfolds	26.5 (12.7)	28.7 (13.1)	-0.90 (-2.21, 0.42)	0.18	-0.76 (-2.05, 0.53)	0.25
Sum of lower skinfolds	25.3(11.8)	27.6 (13.1)	-0.36 (-1.91, 1.19)	0.65	-0.40 (-1.98, 1.18)	0.62
Bioimpedance (Ω)	692.0 (83.1)	688.3 (81.3)	3.33 (-5.23, 11.89)	0.45	3.50 (-5.14, 12.15)	0.43
**Physical activity levels**						
Counts/min	68.7 (33.4)	71.0 (22.9)	-0.15# (-0.34, 0.04)	0.12	-0.18# (-0.36, 0.01)	0.06
MVPA min/24h	49.1 (21.8)	51.1 (20.2)	1.52 (-2.14, 5.17)	0.42	0.51 (-2.97, 3.99)	0.77
**Dietary intake**						
School						
Energy (Kcal)	456.2 (198.8)	488.8 185.2)	-18.83 (-57.54, 19.88)	0.34	-20.56 (-59.82, 18.69)	0.30
Fruit and vegetables (grams)	143.1 (135.0)	93.9 (94.0)	59.88 (34.56, 85.19)	<0.001	3.35 (37.53, 89.17)	<0.001
Sugar (grams)	25.0 (15.7)	29.8 (16.7)	-3.86 (-7.27, -0.45)	0.03	-3.86 (-7.37, -0.36)	0.03

For peer review only

	Intervention group (N=234)	Control group (N=254)	Intervention vs. Control (adjusted for baseline)	p value	Intervention vs. Control (finally adjusted)*	p value
Home						
Energy (Kcal)	1878.7 (1043.6)	1946.8 (957.1)	316.19 (-69.97, 702.34)	0.12	366.81 (-28.14, 761.76)	0.07
Fruit & vegetables (grams)	367.5 (316.6)	342.0 (224.9)	21.61 (-81.26, 124.47)	0.68	18.98 (-89.43, 127.40)	0.73
Sugar (grams)	113.8 (63.7)	121.0 (56.8)	6.88 (-17.04, 30.81)	0.57	9.17 (-15.16, 33.51)	0.45
24h dietary intake						
Energy (Kcal)	2277.2 (1051.7)	2347.2 (901.8)	211.08 (-212.31, 634.48)	0.32	261.08 (-172.88, 695.05)	0.23
Fruit & vegetables (grams)	519.1 (350.2)	446.0 (238.5)	89.64 (-32.51, 211.79)	0.15	86.70 (-42.92, 216.32)	0.19
Sugar (grams)	137.2 (64.2)	150.5 (59.9)	3.16 (-22.74, 29.07)	0.81	4.23 (-22.00, 30.47)	0.75

*Adjusted for sex, ethnicity and baseline values
[#] B calculated for increase of 20 count/min

For peer review only

Discussion

We demonstrated the feasibility of delivering a multicomponent obesity prevention intervention targeting dietary and physical activity behaviours to a socioeconomically disadvantaged, multiethnic population of primary school aged children. The exploratory trial provided an opportunity to refine and modify the programme and yielded important information on acceptability and feasibility of both the intervention and measurements required for assessing outcomes in a definitive RCT.

Strengths and limitations

This is one of few studies focusing on SA populations, which comprise the largest minority ethnic group in the UK, with higher risk of obesity and its consequences. The iterative process of intervention refinement was informed by the MRC framework for complex interventions. Whilst the framework has been used for development of other interventions in NHS settings, we have demonstrated its use in the wider community setting.

The components of the intervention were influenced by stakeholder views and available resources, thus its applicability for wider populations and settings is potentially limited. However, the multifaceted intervention aimed to modify school and family environments and included elements that have been identified as promising in systematic reviews.[7,23] Furthermore the intervention components have theoretical validity for behaviour change in any population, and the incorporated techniques are transferrable. The targeting of South Asian stakeholders for intervention development is likely to have allowed us to exclude intervention components that would not be acceptable to this sub-population. Nevertheless, the developed intervention is likely to be acceptable not only in these ethnic groups, but also in the wider UK population.

Delivery of intervention, undertaken by staff outside the research team, was non-standardised. This allowed a pragmatic approach to be tested, which could be more easily rolled out.

During the trial, all children in schools allocated to the intervention arm were exposed to the intervention components. However, only about half had consent for measurements. We found no significant

For peer review only

differences in sex and ethnicity between consented and non-consented children. Further, the distribution of weight status among children who were measured is similar to national data for this age group[24], suggesting that selection bias was unlikely.

Intervention acceptability and feasibility

A variety of intervention techniques were incorporated with variable success. Environmental restructuring (structured physical activity and play opportunities in school) was feasible and generally accepted. Demonstration of the target behaviour and prompting practice (Villa Vitality, cooking workshops, taster activity sessions and walking groups) had mixed results. Apart from Villa Vitality which was incorporated within the school setting, there was limited participation, despite enthusiasm amongst those who did take part. At a population level, these types of intervention are less feasible to deliver, unless they are incorporated within the school setting. Providing information and prompting identification of role models were feasible and acceptable and would be replicable in a larger trial. Techniques to prompt self monitoring and rewarding successful behaviour were acceptable, but had limited success in this community setting.

During the period of intervention delivery, we used a variety of methods and involved different stakeholders (school staff, parents and children), to assess the acceptability of the intervention components. We also allowed the programme to be modified and the implementation of elements to vary in the different intervention schools. This tailoring to the local school context was critical in determining the success of the intervention. For example in one school, lunchtime supervisors were trained to deliver a structured physical activity programme at lunchtime, but did not go on to deliver the programme. Following this failure of implementation, an enthusiastic teaching assistant was trained, who successfully delivered the intervention. Thus, whilst standardisation of aspects of the intervention is important, some scope for tailoring to local context in terms of implementation and delivery needs to be considered.[25]

For peer review only

Informing a definitive RCT

The intervention was aimed at predominantly SA populations residing in inner city settings. Despite challenges, including language barriers, 80% were successfully followed up. We demonstrated the feasibility of undertaking a wide range of anthropometric measures within school and the feasibility of Actiheart monitors for assessing physical activity in free living children (approximately 90% had usable data). Assessment of dietary intake was less successful at baseline, mainly due to language barriers and difficulties for parents in completing the forms, but the exploratory trial allowed us to refine the administration of the tool, so that measurement was more complete at follow up.

Although the exploratory trial was not powered to examine intervention outcomes, we did find that the direction of effect for most outcomes were in favour of the intervention, supporting the need for a definitive trial. In particular, at follow up children in intervention schools had BMI z-scores on average 0.15kg/m^2 lower than children in control schools, which is in keeping with the effect size reported in a meta-analysis of childhood obesity prevention trials.[7]

Conclusions

We have used the MRC framework for complex interventions to develop a childhood obesity prevention intervention that can be evaluated within the context of a cluster-RCT. Although the intervention was informed by stakeholders, and evidence and guidelines from previous literature, some elements were found not to be feasible or acceptable to participants in practice. The exploratory trial was an essential step in finalising the intervention programme prior to definitive evaluation. Based on the findings from this study, a definitive cluster-RCT is currently underway to assess the clinical and cost-effectiveness of the finalised intervention in primary school children (ISRCTN97000586).

For peer review only

Acknowledgements

S Passmore (Birmingham City Council) advised the study team on identification and engaging of schools and pupils, and the tailoring of interventions to be acceptable to schools.
M Howard (Heart of Birmingham PCT) facilitated the delivery of some of the intervention components through the Primary Care Trust.
E McGee (Birmingham Community Nutrition and Dietetic Service) oversaw the delivery of the cooking workshops and contributed to their evaluation.
K Westgate and S Mayle (MRC Epidemiology Unit, Cambridge) undertook cleaning, reducing and analysing the physical activity data.
C Cleghorn (University of Leeds) contributed to the development of the CADET, oversaw staff training and administration of the tool, and undertook cleaning and analysis of the dietary data.
The sponsor of the study (University of Birmingham) had no role in the study design, data collection, data analysis, data interpretation, writing of the report, or in the decision to submit the paper.

Contributors Statement

Peymané Adab: Dr. Adab conceptualized and designed the study overall, oversaw study planning, delivery and evaluation, wrote the analysis plan and drafted and revised the paper and approved the final manuscript as submitted. She is a guarantor.

Miranda Pallan: Dr. Pallan assisted in the overall delivery of the study, designed the process evaluation, undertook the data cleaning and analysis. She has approved the final manuscript as submitted, and is also a guarantor.

Janet Cade: Professor Cade designed the dietary data assessment tool, oversaw training for researchers collecting data and the analysis of the dietary data obtained. She has approved the final manuscript as submitted.

Ulf Ekelund: Dr. Ekelund advised on physical activity assessment, provided training of researchers in collecting Actiheart data and oversaw the analysis of physical activity data from Actihearts. He has approved the final manuscript as submitted.

Timothy Barrett: Professor Barrett advised on anthropomentric measurement tools used, arranged for training of research staff to undertaken measures and advised on interpretation of the anthropometric data obtained. He has approved the final manuscript as submitted.

Amanda Daley: Dr. Daley advised on physical activity components of the intervention and use of incentives to motivate children. She has approved the final manuscript as submitted.

Jonathan Deeks: Professor Deeks provided statistical support, advised on statistical analysis and on using the data obtained to inform sample size estimation for the definitive trial. He has approved the final manuscript as submitted.

Joan Duda: Professor Duda advised on the psychological measurement instruments, advised research staff on child protection issues and contributed to shaping the physical activity components of the intervention. She has approved the final manuscript as submitted.

Paramjit Gill: Dr. Gill advised on anthropometric measurements to be included, the sampling strategy and on ethnic minority health. He has approved the final manuscript as submitted.

Jayne Parry: Professor Parry advised on process evaluation and contributed to the interpretation of qualitative data obtained. She has approved the final manuscript as submitted.

For peer review only

Raj Bhopal: Professor Bhopal advised on the anthropometric measurements, definition of the target population, tailoring of the intervention to be culturally appropriate and relevant literature on ethnicity and health. He also provided important comments on the final draft and has approved the final manuscript as submitted.

Kar Keung Cheng: Professor Cheng conceived the original idea informing this study, contributed to the planning and delivery. He has approved the final manuscript as submitted.

All authors have contributed to the design of the intervention, advised on study progress and critically revised and approved the final manuscript.

Conflict of Interest Statement

All authors declare there was no support from any organisation for the submitted work; no financial relationships with any organisations that might have an interest in the submitted work in the previous three years; no other relationships or activities that could appear to have influenced the submitted work. Amanda Daley is supported by an NIHR Senior Research Fellowship award. The other authors have no disclosures relevant to this article. The views expressed in this publication are those of the authors and not necessarily those of the NHS, The National Institute for Health Research or the Department of Health. Primary Care Clinical Sciences is a member of the NIHR National School for Primary Care Research. The funding organisation did not play any role in design and conduct of the study; collection, management, analysis, and interpretation of the data; and preparation, review, or approval of the manuscript.

Data Sharing Statement

The BEACHeS study dataset is available on request from the study investigators.

Funding source

The Birmingham healthy Eating, Active lifestyle for Children Study (BEACHeS) was funded by the National Prevention Research Initiative (NPRI, http://www.npri.org.uk), Grant no. G0501292 and we are grateful to all the funding partners for their support: British Heart Foundation; Cancer Research UK; Department of Health; Diabetes UK; Economic and Social Research Council; Medical Research Council; Research and Development Office for the Northern Ireland Health and Social Services; Chief Scientist Office, Scottish Executive Health Department; Welsh Assembly Government and World Cancer Research Fund.

Clinical Trial Registration: ISRCTN51016370

Licence for Publication Statement

"I Miranda Pallan, the Corresponding Author of this article contained within the original manuscript which includes any diagrams & photographs, other illustrative material, video, film or any other material howsoever submitted by the Contributor(s) at any time and related to the Contribution ("the Contribution"), have the right to grant on behalf of all authors and do grant on behalf of all authors, a licence to the BMJ Publishing Group Ltd and its licensees, to permit this Contribution (if accepted) to be published in BMJ Open and any other BMJ Group products and to exploit all subsidiary rights, as set out in the licence at: (http://group.bmj.com/products/journals/instructions-for-authors/BMJOpen_licence.pdf)

For peer review only

References

(1) Lobstein T, Baur L, Uauy R. Obesity in children and young people: a crisis in public health. Obes Rev 2004; **5**(Suppl. 1):4-85.

(2) Baker JL, Olsen LW, Sorensen TIA. Childhood Body-Mass Index and the risk of coronary heart disease in adulthood. N Engl J Med 2007; **357**:2329-37.

(3) Stamatakis E, Wardle J, Cole TJ. Childhood obesity and overweight prevalence trends in England: evidence for growing socioeconomic disparities. Int J Obes 2010; **34**:41-7.

(4) NHS Information Centre for Health and Social Care. National Child Measurement Programme: England, 2011/12 school year. The Health and Social Care Information Centre, 2012.

(5) Dinsdale H, Ridler C, Rutter H. National Child Measurement Programme: Changes in children's body mass index between 2006/7 and 2010/11. Oxford, National Obesity Observatory, 2012.

(6) Summerbell CD, Waters E, Edmunds LD, et al. Interventions for preventing obesity in children. Cochrane Database of Systematic Reviews 2005, Issue 3, John Wiley & Sons, Ltd Chichester, UK, DOI: 10 1002/14651858 CD001871 pub2.

(7) Waters E, de Silva-Sanigorski A, Hall B J, et al. Interventions for preventing obesity in children. Cochrane Database of Systematic Reviews 2011, Issue 12, John Wiley & Sons, Ltd Chichester, UK, DOI:10 1002/14651858 CD001871 pub3.

(8) McPherson K, Marsh T, Brown M. Tackling obesities: future choices - modelling future trends in obesity and the impact on health. Report No. 2. London, Government Office for Science, 2007.

(9) Wang Y, Wu Y, Wilson RF, et al. Childhood obesity prevention programs: comparative effectiveness review and meta-analysis. Comparative Effectiveness Review No. 115. Rockville, MD, Agency for Healthcare Research and Quality, 2013.

(10) National Institute for Health and Clinical Excellence. Obesity: the prevention, identification, assessment and management of overweight and obesity in adults and children. London, 2006.

(11) Campbell M, Fitzpatrick R, Haines A, et al. Framework for design and evaluation of complex interventions to improve health. BMJ 2000; **321**:694-6.

(12) Pallan M, Parry J, Cheng KK, Adab P. Development of a childhood obesity prevention programme with a focus on UK South Asian Communities. Prev Med 2013; **57**:948-54.

(13) Varni JW, Limbers CA, Burwinkle TM. How can young children reliably and validly self-report their health-related quality of life?: an analysis of 8,591children across age subgroups with the PedsQL 4.0 generic core scales. Health Qual Life Outcomes 2007; **5**:1.

(14) Marsh HW, Barnes J, Cairns L, Tidman M. Self-Description Questionnaire: Age and sex effects in the structure and level of self-concept for preadolescent children . J Educ Psychol 1984; **76**:940-56.

(15) Harter S, Pike R. The pictorial scale of perceived competence and social acceptance for young children. Child Dev 1984; **55**:1969-82.

For peer review only

(16) Collins ME. Body figure perceptions and preferences among preadolescent children. Int J Eat Disord 1991; **10**:199-208.

(17) Cade JE, Frear L, Greenwood DC. Assessment of diet in young children with an emphasis on fruit and vegetable intake: using CADET – Child and Diet Evaluation Tool. Public Health Nutr 2006; **9**:501-8.

(18) Brage S, Brage N, Franks P, et al. Reliability and validity of the combined heart rate and movement sensor Actiheart. Eur J Clin Nutr 2005; **59**:561-70.

(19) Pallan M., Parry J., Adab P. Contextual influences on the development of obesity in children: a case study of UK South Asian communities. Prev Med 2012; **54**:205-11.

(20) Michie S, Ashford S, Sniehotta FF, et al. A refined taxonomy of behaviour change techniques to help people change their physical activity and healthy eating behaviours: the CALO-RE taxonomy. Psychol Health 2011; **26**:1479-98.

(21) Cole TJ, Freeman JV, Preece MA. Body mass index reference curves for the UK, 1990. Arch Dis Child 1995; **73**:25-9.

(22) Corder K, Brage S, Wareham NJ, et al. Comparison of PAEE from combined and separate HR and movement models in children. Med Sci Sports Exerc 2005; **37**:1761-7.

(23) Lavelle HV, Mackay DF, Pell JP. Systematic review and meta-analysis of school-based interventions to reduce body mass index. J Public Health 2012; **34**:360-9.

(24) National Obesity Observatory. National Child Measurement Programme: detailed analysis of the 2007/08 national dataset. London, 2009.

(25) Hawe P, Shiell A, Riley T. Complex interventions: how "out of control" can a randomised controlled trial be? BMJ 2004; **328**:1561-3.

For peer review only

Figure 1: Flow diagram of recruitment and follow up of participants in the exploratory trial

For peer review only

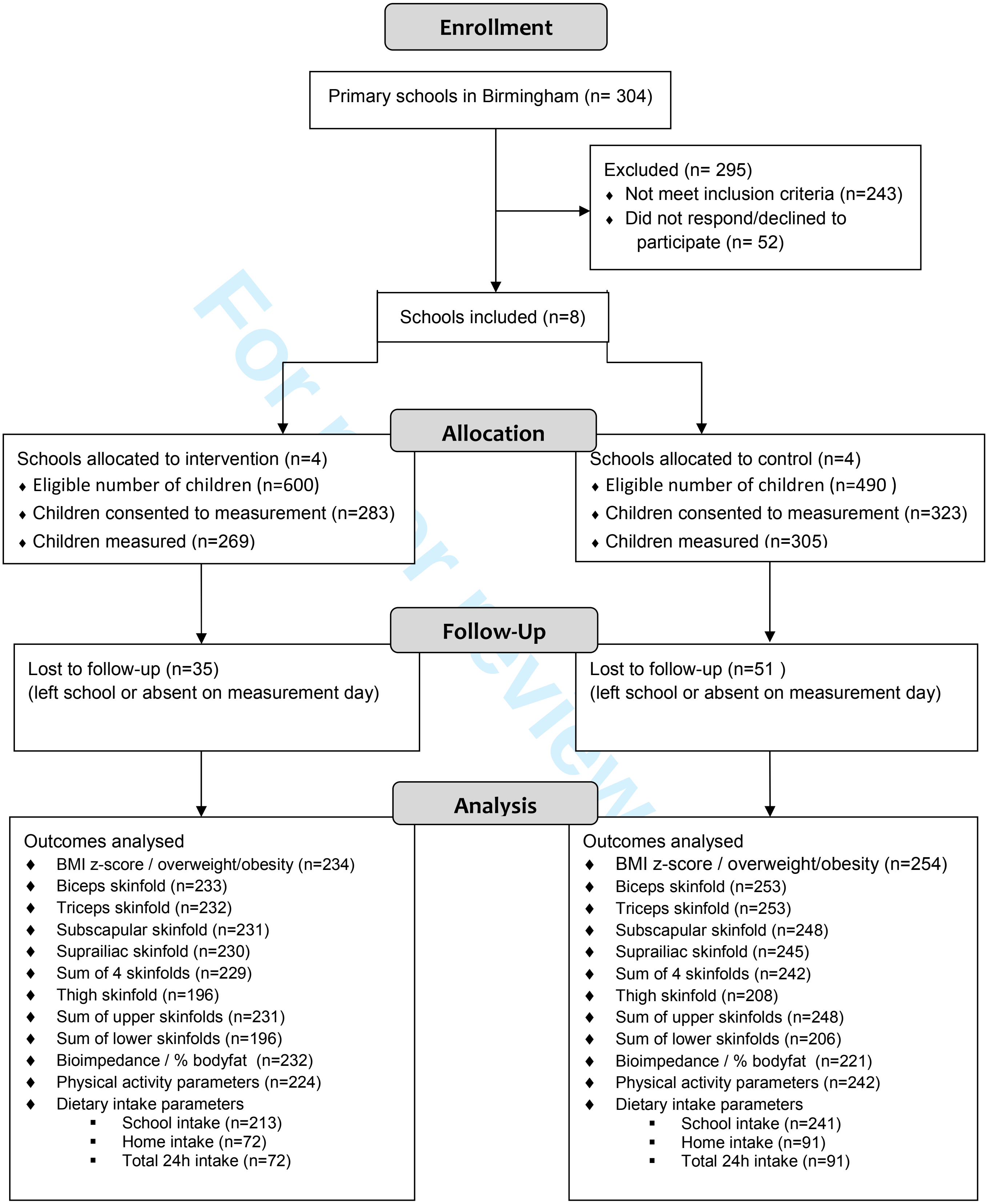
Enrollment
Primary schools in Birmingham (n= 304)
Excluded (n= 295)
♦ Not meet inclusion criteria (n=243)
♦ Did not respond/declined to participate (n= 52)
Schools included (n=8)
Allocation
Schools allocated to intervention (n=4)
♦ Eligible number of children (n=600)
♦ Children consented to measurement (n=283)
♦ Children measured (n=269)
Schools allocated to control (n=4)
♦ Eligible number of children (n=490)
♦ Children consented to measurement (n=323)
♦ Children measured (n=305)
Follow-Up
Lost to follow-up (n=35)
(left school or absent on measurement day)
Lost to follow-up (n=51)
(left school or absent on measurement day)
Analysis
Outcomes analysed
♦ BMI z-score / overweight/obesity (n=234)
♦ Biceps skinfold (n=233)
♦ Triceps skinfold (n=232)
♦ Subscapular skinfold (n=231)
♦ Suprailiac skinfold (n=230)
♦ Sum of 4 skinfolds (n=229)
♦ Thigh skinfold (n=196)
♦ Sum of upper skinfolds (n=231)
♦ Sum of lower skinfolds (n=196)
♦ Bioimpedance / % bodyfat (n=232)
♦ Physical activity parameters (n=224)
♦ Dietary intake parameters
▪ School intake (n=213)
▪ Home intake (n=72)
▪ Total 24h intake (n=72)
Outcomes analysed
♦ BMI z-score / overweight/obesity (n=254)
♦ Biceps skinfold (n=253)
♦ Triceps skinfold (n=253)
♦ Subscapular skinfold (n=248)
♦ Suprailiac skinfold (n=245)
♦ Sum of 4 skinfolds (n=242)
♦ Thigh skinfold (n=208)
♦ Sum of upper skinfolds (n=248)
♦ Sum of lower skinfolds (n=206)
♦ Bioimpedance / % bodyfat (n=221)
♦ Physical activity parameters (n=242)
♦ Dietary intake parameters
▪ School intake (n=241)
▪ Home intake (n=91)
▪ Total 24h intake (n=91)

Preventing childhood obesity, exploratory trial focusing on South Asians: BEACHeS

Appendix: Detailed findings from the process evaluation of intervention components included in the BEACHeS programme

Intervention component		Evaluation findings
**School based activities**	Physical activities within school day	***"Wake Up Shake Up":*** 3 intervention schools implemented this to varying degrees (twice a day in one school, once or less in others).<br>Participation by children was greater if session was compulsory rather than optional.<br>Questionnaire responses from the children suggested that it was generally well received, although a few children were reluctant to join in.<br>Staff were generally enthusiastic about running this, but identified lack of time and lack of space as potential barriers to success.<br>*"[Wake Up Shake Up is] really popular, going really well, parents are now starting to join in a lot more because the idea was that the parents would join in as well. So I am really pleased with how that's coming on"* (teacher and school physical education lead)<br>***Organised playground activities:*** All 4 schools received training and attempted to introduce this at lunch times. Implementation varied greatly between schools, with some schools not consistently being able to deliver.<br>A key factor for successful implementation was having an enthusiastic member of staff to inspire the play leaders. The school that had the least success with implementation was where the lunchtime supervisors were trained as play leaders but did not have a member of staff to lead them.<br>Provision of playground equipment and using a zoning system in the playground supported successful delivery.<br>Children and staff indicated that they enjoyed the scheme and supported it.<br>*"What I have found with [a teaching assistant] going out, who's doing like the parachute games and that with them is much better, because the dinner ladies just didn't take it on board. I think they came to the training because they had to but it's had no impact, I've got to be honest"* (deputy head teacher)<br>***Take 10 curricular materials:*** 2 of the schools implemented this component.<br>Success depended on the enthusiasm and motivation of the class teachers. Teachers who used Take 10 were keen to continue and expand its use.<br>Some members of staff identified lack of training and familiarity with the material as barriers to its implementation.<br>*"I know it [Take 10] works really well, I know it's really accessible....the only problems I have is with the staff doing it or not doing it"* (teacher and school physical education lead)
	Incentive scheme to encourage physical activity out of school	The major obstacle to the success of this component was retention of leisure venues and maintenance of enthusiasm.<br>Most venues that were initially recruited no longer actively participated after 2-3 months. Staff turnover, change in management, high frequency of temporary staff, low perceived importance and lack of interest from leisure centre staff contributed to the failure of this component.<br>School staff felt that incentive schemes were generally a good way of motivating children. However, in addition to problems with venue participation, other problems were identified. Teachers felt that motivation needed regular reinforcement (e.g. by class teachers), children

For peer review only

		needed a specific goal to work towards, and a shorter time frame (e.g. one term rather than one school year) and tangible reward (named item, rather than an unspecified prize) would have helped to focus the children.<br>Children were generally enthusiastic about collecting stickers. However difficulties experienced with receiving stickers from the majority of venues de-motivated them.<br>Parents had found information on local sporting and leisure venues useful. The majority of parents were also in favour of incentive schemes to encourage children to undertake physical activities.<br>*“they [the children] were so eager to collect the stickers... especially at the beginning when we were having trouble with the stickers, when they [the leisure venues] weren’t giving them”* (deputy head teacher)
	Attendance at a course run by a Premier league football club	This component was extremely popular both with children and school staff, with high (near 100%) attendance<br>Most parents did not recall that their children attended the ‘Villa Vitality’ day<br>Children attending Villa Vitality reported significantly greater levels of physical activity outside of school time 6-weeks post attendance, compared to control children.<br>*“they really enjoyed that [Villa Vitality], I think that was... you know, once they got into it and realised what it was about and that they got a lot out of it, then they really enjoyed it”* (deputy head teacher)
**Increasing skills of families through activity-based learning**	Cooking courses for family members	Uptake was generally lower than capacity (4-10 participants / course). Uptake was highest in one school with an enthusiastic parent link worker.<br>There was some drop out over the 5 weeks of the course but over 90% of participants attended 3 or more sessions.<br>The courses were well received by those who attended. Compared to parents who did not participate, participants had lower baseline confidence in their cooking ability. Following the course, participants had higher levels of reported confidence than at baseline in shopping for healthy food and cooking healthy meals. They also reported that the family was eating more healthily.<br>Courses where the children were also invited to cook with their parents were generally better attended. The model of parents learning about healthy cooking with their children was popular with parents, children and school staff.<br>School staff thought it was feasible to use school time for healthy cooking sessions for children where parents could be invited along.<br>Supporting information such as healthy eating tips and recipes in newsletters was well received by parents.<br>*“[cooking courses were] very, very successful, wonderful way of doing it. So the parents were learning about healthy ways of cooking and so on without it looking as if they were having a finger wagged at them”* (teacher and school-community liaison manager)
	Information on local leisure opportunities and week-end “taster” sessions for families	Uptake of the taster activity sessions was generally poor, although it did increase through the year through recommendations by those who attended.<br>Activities were very well received by those who did attend.<br>School staff were supportive by the taster activity sessions and expressed surprise and disappointment by the low uptake rates.<br>The taster sessions were extremely resource intensive to run, in terms of cost and staff time.<br>Most parents (>90%) found the information signposting healthy activities and venues useful, and school staff also felt this had been useful for

For peer review only

		parents and served to motivate them to try out local facilities.<br>*"I was so disappointed that out of 120 children, so few families took up these free visits when they were actually picked up and taken somewhere"* (deputy head teacher)
	Training walk leaders to initiate community walking programmes	This component proved unfeasible, as there was a lack of volunteers to train as walk leaders. Even those who expressed an initial interest failed to attend the training. Despite repeated efforts to recruit community volunteers, only one person attended a training session, but they failed to organise any walking trips.<br>*"we put signs up and sent leaflets out to see if anybody was interested in being a walk leader or training and I don't think we got anybody back at all"* (deputy head teacher)

For peer review only

BMJ Open

Preventing childhood obesity, phase II feasibility study focusing on South Asians: BEACHeS

Journal:	*BMJ Open*
Manuscript ID:	bmjopen-2013-004579.R1
Article Type:	Research
Date Submitted by the Author:	12-Mar-2014
Complete List of Authors:	Adab, Peymane; The University of Birmingham, Public Health, Epidemiology & Biostatistics<br>Pallan, Miranda; University of Birmingham, Public Health, Epidemiology & Biostatistics<br>Cade, Janet; University of Leeds,<br>Ekelund, Ulf; MRC Epidemiology Unit,<br>Barrett, Timothy; Birmingham Childrens HospitalUniversity of Birmingham, Diabetes Unit Institute of Child Health<br>Daley, Amanda; University of Birmngham, Primary Care Clinical Sciences<br>Deeks, Jon; University of Birmingham, Public Health, Epidemiology & Biostatistics<br>Duda, Joan; University of Birmingham, Sport, Exercise & Rehabilitation Sciences<br>Gill, Paramjit; University of Birmingham, Primary Care Clinical Sciences<br>Parry, Jayne; University of Birmingham, Public Health, Epidemiology & Biostatistics<br>Bhopal, Raj; University of Edinburgh<br>Cheng, Kar Keung; The University of Birmingham, Public Health, Epidemiology & Biostatistics
**Primary Subject Heading**:	Public health
Secondary Subject Heading:	Public health
Keywords:	Obesity, Children, Prevention

SCHOLARONE™ Manuscripts

Title: Preventing childhood obesity, phase II feasibility study focusing on South Asians: BEACHeS

Authors: Peymané Adab (MD)[1], Miranda Pallan (PhD)[1], Janet Cade (PhD)[2], Ulf Ekelund (PhD)[3], Timothy Barrett (PhD)[4], Amanda Daley (PhD)[1], Jonathan Deeks (PhD)[1], Joan Duda (PhD)[5], Paramjit Gill (DM)[1], Jayne Parry (MD)[1], Raj Bhopal (PhD)[6], KK Cheng (PhD)[1]

Affiliations:
[1]School of Health & Population Sciences (Public Health, Epidemiology and Biostatistics and Primary Care Clinical Sciences), University of Birmingham
[2]Nutritional Epidemiology Group, School of Food Science and Nutrition, University of Leeds
[3]MRC Epidemiology Unit, Institute of Metabolic Science, Cambridge & Department of Sport Medicine, Norwegian School of Sport Sciences
[4]School of Clinical and Experimental Medicine, University of Birmingham
[5]Sport & Exercise Sciences, The University of Birmingham
[6]Edinburgh Ethnicity and Health Research Group, Centre for Population Health Sciences, University of Edinburgh

Address correspondence to:
Miranda J Pallan, Public Health, Epidemiology and Biostatistics, University of Birmingham, Edgbaston, Birmingham, B15 2TT, UK.
Tel: +441214147990
Fax: +441214147878
E-mail: m.j.pallan@bham.ac.uk

Key words: Childhood obesity prevention; feasibility study; MRC framework for complex interventions

Word count:
Abstract: 273
Manuscript: 3743

For peer review only

Abstract

Objectives: To assess feasibility and acceptability of a multifaceted, culturally appropriate intervention for preventing obesity in South Asian children, and to obtain data to inform sample size for a definitive trial.

Design: Phase II feasibility study of a complex intervention.

Setting: Eight primary schools in inner city Birmingham, UK, within populations that are predominantly South Asian.

Participants: 1090 children aged 6-8 years took part in the intervention. 571 (85.9% from South Asian background) underwent baseline measures. 85.5% (n=488) were followed up 2 years later.

Interventions: The one-year intervention consisted of school and family based activities, targeting dietary and physical activity behaviours. The intervention was modified and refined throughout the period of delivery.

Main outcome measures: Acceptability and feasibility of the intervention and of measurements required to assess outcomes in a definitive trial. The difference in BMI z-score between arms was used to inform sample size calculations for a definitive trial.

Results: Some intervention components (increasing school physical activity opportunities, family cooking skills workshops, signposting of local leisure facilities and attending day event at a football club) were feasible and acceptable. Other components were acceptable, but not feasible. Promoting walking groups was neither acceptable nor feasible. At follow up, children in the intervention compared with the control group were less likely to be obese (OR 0.41; 0.19 to 0.89), and had lower adjusted BMI z-score [-0.15 (-0.27, -0.03)].

Conclusions: The feasibility study informed components for an intervention programme. The favourable direction of outcome for weight status in the intervention group supports the need for a definitive trial. A cluster randomised controlled trial is now underway to assess the clinical and cost-effectiveness of the intervention.

For peer review only

Article Summary

Strengths and limitations of the study

- We report the findings of a feasibility study of a childhood obesity prevention intervention that focuses on primary school-aged children from South Asian communities in the UK. Despite their susceptibility to the cardiometabolic consequences of obesity, little obesity prevention research has been undertaken in these communities previously.
- The early phases of the UK Medical Research Framework for complex health interventions has guided the intervention development and evaluation process undertaken in this feasibility study.
- The feasibility and acceptability of the childhood obesity prevention intervention components was variable and context dependent, however, the exploratory nature of the study enabled us to modify and refine delivery of the intervention throughout.
- Development and evaluation of the feasibility and acceptability of the intervention was undertaken in materially disadvantaged, predominantly South Asian communities, thus its transferability would be dependent on tailoring to the specific local context.
- The final intervention programme, following modification and refinement in this feasibility study, is being definitively evaluated in an ongoing cluster-randomised controlled trial.

For peer review only

Introduction

Childhood obesity is a growing problem worldwide.[1] Apart from psychological and social problems, longitudinal studies show adverse future health consequences in children as young as 7 years old who are obese.[2] In the UK, although childhood overweight prevalence has stabilised, socioeconomic disparities have widened, with increasing trend in more deprived sub-populations.[3] Data from the national childhood surveillance programmes in England show that at school entry (age 4-5 years), 9.5% of children are obese (i.e., above 95th percentile for national reference standards), but this prevalence doubles (19.2%) by the end of primary school (age 11).[4] The rate of increase among children from South Asian (SA) ethnic groups, especially girls, is greater than that for the population as a whole (increasing trend of 1.13% and 0.66% per year for Bangladeshi and Pakistani girls respectively, compared with 0.35% yearly increase in White British).[5] Thus the primary school period presents a key phase for prevention, and SA are an important target group.

However, despite numerous systematic reviews,[6,7] reports[8,9] and guidelines,[10] evidence for effective approaches to prevention is limited, particularly among minority ethnic groups. Relevant trials suggest that multifaceted school-based interventions have potential, particularly those that also include a home or community element, but the most effective combination of components is not clear.[7,9] The need for involving stakeholders, such as families, schools and local communities, in the decision making regarding potential intervention strategies has been highlighted.[6] Furthermore, for a complex intervention such as obesity prevention, which has several interconnecting components, a rigorous and iterative phased approach is required to improve study design, execution and applicability of results. The UK Medical Research Council (MRC) proposed a framework for such interventions.[11] Given the growing problem of obesity and lack of clarity on effective approaches to

For peer review only

prevention, it would be unwise to embark on another trial without thorough attention to the early phases described in the MRC framework.

The Birmingham healthy Eating and Active lifestyle for CHildren Study (BEACHeS http://www.birmingham.ac.uk/research/activity/mds/projects/HaPS/PHEB/WAVES/BEACHeS/index.aspx), used the theoretical and modelling phases of the MRC framework to develop a multifaceted childhood obesity prevention programme, targeting SA children (phase I).[12] Here we report on the feasibility study (phase II). The aim was to assess feasibility and acceptability of the intervention. In addition we wanted to obtain data to inform a definitive (phase III) cluster-randomised controlled trial (RCT).

For peer review only

Materials and Methods

The feasibility study was conducted in eight Birmingham primary schools from 2006 to 2009. Children underwent baseline measures between December 2006 and June 2007. Four schools were selected to receive the intervention (2007/8 academic year), and the remainder had no active intervention. Follow up data were collected two years after baseline.

Setting

Birmingham is UK's second city with a high minority ethnic population (34%), one fifth being from the three main SA communities (Pakistani, Bangladeshi and Indian). We obtained a list of all Local Authority-maintained primary schools in Birmingham. Of 304 schools, 52 had ≥50% of pupils from SA background (mean 75%). These, compared with the remainder, had a higher proportion of children eligible for free school meals (FSM), indicating higher deprivation. Schools were ranked in order of FSM eligibility, and those from either extreme were successively invited until 8 agreed to take part.

Participants

Pupils from years 1 and 2 (aged 5-7 years) were invited to participate. Parents of the children were approached by letter distributed through the schools, and active opt-in consent was sought for their child to participate in measurements.

Baseline and follow up measures

Age, sex and ethnicity data (from parent report at school entry) were obtained from school records on all eligible children in participating schools. Children with consent also underwent a range of anthropometric measurements, including standing height (measured to nearest 0.1cm with a Leicester Height Measure), weight (measured to nearest 0.1 kg with aTanita bioimpedance monitor), two measures of waist circumference (measured to nearest 0.5cm),

For peer review only

and skinfold thickness at five sites (biceps, triceps, subscapular, suprailiac and thigh; measured using a Holtain calliper). Children also completed interviewer administered questionnaires (not discussed in this paper, but including: quality of life (PedsQL),[13] self concept (Marsh self-description questionnaire),[14] perceived physical competence (Harter Pictorial Scale for Young Children),[15] and body image perception (adapted Collin's Pictorial Image Scale)[16]). All measures were undertaken by trained researchers using standard protocols.

Dietary intake was assessed using the Child And Dietary Evaluation Tool (CADET)[17]; a 24-hour food tick list that has been validated against a semi-weighed diary in children aged 3-7 years. A researcher completed the CADET for children during school hours, and parents were given instructions for completing it for the remainder of the 24 hour period. Physical activity levels were assessed using the Actiheart monitor (CamnTech, Papworth UK) worn for five consecutive days, including a weekend. This is validated for use in children[18] and was set up to measure acceleration and heart rate at 30 second epochs. In addition, parents were asked to complete questionnaires which included questions on family composition, and family dietary and physical activity habits.

Intervention

The process for intervention development has been reported elsewhere,[12] but in brief, the multicomponent intervention was developed by combining evidence from the literature with views from key stakeholders drawn from SA communities (including parents, teachers, school nurses, dieticians, community leaders, school governors, and retail and leisure representatives close to schools) and a multi-disciplinary group of relevant professionals. Important contextual data were gained from stakeholders, which was critical for informing intervention development and highlighted potential barriers (e.g. cultural unacceptability of certain types of physical activity for girls), as well as opportunities for intervention (e.g.

For peer review only

schools being considered a natural environment for providing skills to families), in relation to SA communities.[18,19] A review of local facilities, resources and opportunities related to healthy eating and the promotion of physical activity targeting children was used to inform the design and encourage longer term sustainability of the intervention. We also took account of national childhood obesity prevention policy during the development process to try and ensure that the intervention had an impact that was additional to existing national initiatives. The intervention targeted both diet and physical activity behaviours and consisted of two main strands: i) increasing children's physical activity levels and promoting healthy eating through schools, and ii) increasing skills among family members through family educational activities. A number of intervention techniques (as defined in the CALO-RE Taxonomy of behaviour changed techniques for physical activity and healthy eating[20]) were utilised to deliver each intervention component. A more detailed description of the intervention is provided in Table 1.

For peer review only

Table 1: Intervention components and techniques[20] included in the BEACHeS intervention programme and findings from the process evaluation

Intervention component	Aim	Intervention techniques	Description	Agent responsible for delivery	Evaluation method	Evaluation findings
			School based activities			
Physical activities within school day	To increase the amount of time that children are physically active within the school day	Environmental restructuring<br>Prompt practice	Three elements introduced into schools:<br>1. 'Wake Up Shake Up': a short (10 minutes) organised daily dance or exercise routine to music<br>2. Organised playground activities at lunch and break times through the training of school staff to act as "play leaders"<br>3. 'Take 10': teaching resource which links 10 minutes physical activity in the classroom to curricular subjects.	Trained school staff (including teachers, teaching assistants or lunch time assistants). The decision of which staff members to train for this component took into account individual school circumstances and was made in consultation with each school.	• Interviews with school staff<br>• Observation of sessions in schools<br>• Self-completion questionnaires administered to children and parents	Overall, school staff with a responsibility for health were enthusiastic and committed to introducing these schemes, and all schemes were acceptable to children. Individual school and staff factors strongly influence the success of each element in the different schools. Parents, in general felt that the amount of physical activity their children were undertaking in school had increased over the last year.
Incentive scheme to encourage physical activity out of school	To increase the amount of time outside of school hours that children spend doing leisure physical activities	Prompt self monitoring of behaviour<br>Prompt practice<br>Provide rewards contingent on successful behaviour	Children received a sticker collection card from school and information on local participating sports and leisure venues. Each time a child attended a venue, they collected a sticker. The child with the most stickers in each school received a prize.	Sticker collection card delivered through school class teacher. Stickers handed out by staff at leisure venues.	• Interviews with school staff<br>• Telephone survey of leisure venue staff<br>• Assessment of returned collection cards<br>• Questionnaires to children	Although this type of incentive scheme appears acceptable to children, parents and school staff alike, it was not feasible in terms of maintaining cooperation of participating venues. An element that was well received and could be retained, is the signposting information given to children and families.
Attendance at a course run by a Premier league	To encourage physical activity and healthy eating through	Provide information on consequences of behaviour<br>Model/demonstrate	School classes attend a 'Villa Vitality' day. Half the day is spent with Football Club coaches, exercising and learning football	Aston Villa Football Club Community programme staff deliver on day of visit to club.	• Interviews with school staff<br>• Self-completion questionnaires to parents	This was highly acceptable to children and school staff and is feasible to deliver to the target age group. There is some evidence that it may favourably alter children's health-

For peer review only

football club	an iconic sporting institution	behaviour<br>Prompt identification of role model/advocate<br>Goal setting (behaviour)	skills, and the other half of the day is an interactive learning session on healthy eating and healthy lifestyles. Teachers provided with material to deliver over 6 weeks to reinforce messages, and encourage weekly challenges or goals.	School class teachers deliver 6 weekly lessons after club visit.	• Pre- and 6-week post intervention questionnaires (knowledge, attitudes & behaviour) administered to children	behaviours.
**Increasing skills of families through activity-based learning**						
Cooking courses for family members	To increase healthy cooking skills and confidence and influence dietary behaviour	Provide information on consequences of behaviour in general<br>Model/demonstrate behaviour<br>Provide instruction on how to perform the behaviour<br>Prompt generalisation of behaviour	Five week courses on healthy cooking were delivered through schools to parents or other family members, some courses include children. Courses ran successively to allow all parents to attend if they wanted. Healthy recipes were distributed to support the course content.	Birmingham Community NHS Trust dietetics staff.	• Interviews with school staff<br>• Uptake rates for courses<br>• Participant pre and post-course questionnaires	This component was popular with those who participated and there was some evidence that it influenced confidence and cooking practices. Running sessions for parents and children was the most popular model, and having the sessions based in school time for children and inviting parents to attend improved attendance.
Information on local leisure opportunities and “taster” sessions for families	To equip families with the knowledge and skills to undertake physical activities with their children in their leisure time	Provide information on when and where to perform behaviour<br>Model/demonstrate behaviour	Parents were given information on local sporting and leisure venues and events. They were invited to attend weekend taster sessions with their children, through school. Activities range from cricket and football, to archery, climbing and dry-slope skiing. There was no cost for the activities and transport was provided.	BEACHeS research staff compiled list of venues and prepared signposting sheets. BEACHeS research staff accompanied families to leisure venues, where leisure venue staff delivered sessions.	• Interviews with school staff<br>• Uptake of the taster sessions<br>• Self-completion questionnaires to parents and children	This component was resource intensive to deliver, and uptake was very low. However, the signposting information was used by families, and was appreciated.

For peer review only

Training walk leaders to initiate community walking programmes	To increase walking by families and other community members through organised leisure walks	Model/demonstrate behaviour<br><br>Prompt practice	Community volunteers were recruited through schools to become trained walk leaders. Training was provided to equip volunteers to organise and lead walks in their local community.	Walk-leader training programme delivered through Heart of Birmingham NHS Trust.	• Assessment of numbers attending training to be walk leaders<br>• Monitoring numbers joining walking groups	This component proved unfeasible, as there was a lack of volunteers. Even those who expressed an initial interest failed to attend the training. Despite repeated efforts to recruit community volunteers, no one attended the training in any of the four BEACHeS intervention communities.

For peer review only

Allocation of intervention

This was a non-randomised feasibility trial. After baseline measurements were completed, schools were allocated to intervention or control arms. We matched schools by size, and proportion of children eligible for FSM. We then took the geographical location of the schools into account and allocated the matched pairs to either the intervention or control arm so that we minimised the chance of contamination between the two arms.

Process measures

The main aim of the study was to assess intervention feasibility and acceptability. Each component was evaluated separately, using a variety of methods. These included collection of uptake data, direct observation, questionnaires to children and parents and interviews with key school staff. The questionnaires were also used to evaluate overall perceptions of the intervention and engagement with different intervention components. Topics covered in the semi-structured interviews included exploration of how the different intervention components were implemented, which elements were perceived to work well and ideas for further development. The interviews were tape-recorded, transcribed and analysed thematically.

Other measures and analysis

We assessed the feasibility of obtaining outcome data, primarily body mass index (BMI), but also diet and physical activity and other anthropometric measures as described above. Exploratory comparison between intervention and control children was also undertaken to determine effect size.

Height and weight data were used to calculate BMI (kg/m^2) and converted into standard deviation scores (BMI z-score) using the UK 1990 growth reference charts.[21] Children were categorised as underweight, healthy weight, overweight or obese using the 2nd, 85th and 95th centile cut-offs. For waist circumference and skinfolds, the mean was used for analyses.

For peer review only

Skinfold measures were combined to obtain sum, upper (biceps, triceps and subscapular) and lower (suprailiac and thigh) skinfolds.

Data from the CADET were coded and analysed by a food diary analysis program (DANTE, University of Leeds) to estimate total energy intake (KJ) and amount of fruit and vegetables, and sugar consumed. Data on foods consumed in school and at home were analysed separately, then combined to obtain estimates for the complete 24 hour measurement period.

Accelerometry data were used to assess physical activity levels (http://www.mrc-epid.cam.ac.uk/Research/Programmes/Programme_5/InDepth/Programme%205_Downloads.html). Total daily volume of physical activity was estimated and expressed as average counts per minute (cpm). The mean duration of daily moderate to vigorous physical activity (MVPA, min/day) was calculated (400 cpm cut-off for lower threshold).[22] The proportion participating in ≥60 minutes MVPA (as recommended by international guidelines) was also calculated.

Statistical analyses were undertaken using STATA (v11). The intraclass correlation coefficient (ICC) for the main outcome (BMI z-score) was calculated, but because of the small number of schools, clustering was not taken into account for the analysis. We analysed final BMI, diet and physical activity levels of children in the intervention, compared with those in the control group. To adjust for baseline differences, we initially developed multiple linear regression models, which included the relevant baseline values of BMI, dietary factors or physical activity measures as covariates. Further models were then developed which also included potential confounders as covariates (age, sex, ethnicity). Logistic regression was used to assess risk of obesity (compared with all non-obese children), and likelihood of meeting ≥60 minutes MVPA at follow up in the intervention, compared with control children.

Ethical approval

For peer review only

Ethical approval was granted by The Black Country Research Ethics Committee (08/H1202/22). Approval was sought for active consent from parents for the child measurements, whilst consent for participation in the intervention was at school level.

For peer review only

Results

Feasibility and acceptability of intervention components

Some intervention components (particularly those delivered through school) were more successfully delivered than others. Intervention was modified during the course of delivery to optimise participation and in response to feedback. The findings are summarised in Table 1, and details are reported in the appendix.

Two intervention components were found to be unsuitable to include within an intervention programme in the format delivered. First was the scheme to incentivise out of school leisure activities. Poor co-operation from leisure venues and lack of resources to continually remind and motivate children contributed to the failure of this component. Second was the training of walk leaders. Despite effort to recruit through school staff, influential parents and various forms of publicity, volunteers were not forthcoming. The only person who underwent training did not undertake any walking groups.

One component was partially successful. Signposting of leisure facilities in the local area was popular among parents and school staff. However, attendance at organised taster sessions was poor, which was outweighed by the high staff and monetary resources required to deliver the component. The taster sessions were therefore not feasible to include in a larger trial.

The other intervention components had varying degrees of success, and the process evaluation highlighted how delivery could be improved. Individual school characteristics and differences between staff members strongly influenced the success of each element in the different schools.

For peer review only

Acceptability of allocation to control

Acceptability of non-intervention was assessed through interviews with control school staff. All understood the need for a control arm. One would have liked alternative support to compensate for not being offered the intervention. In other control schools, staff expressed that being part of the study had benefitted them in other ways, and contributed to the school's status as a 'healthy school'.

Outcomes

Feasibility of outcome measures

There were 1090 eligible children in the eight participating schools (range 54-180). Of these, 606 (55.6%) had parental consent and anthropometric measures were completed on those in school on measurement days (n=571, 94.2% of consented). Useable data (≥3 days) for Actiheart were available for 508 (89.0%). Completion of CADET was more variable. Although 445 (77.9%) were returned at baseline, 269 (47.1%) were complete, of which two thirds (n=174) had usable data. Two years after the baseline measures, 488 children (85.5%) were successfully followed up. The proportion with usable Actiheart data was similar to baseline. However a higher proportion (n=454, 93%) had a completed CADET, although only 163 (36%) had usable home data.

Findings from the feasibility study

A total of 574 children were included in the trial (Figure 1), of whom 85.9% were SA. Baseline characteristics are summarised in Table 2 (anthropometric measures were completed for 571 of the 574 participating children). The age, sex and ethnicity of those who took part were similar in distribution to the characteristics of the non-consented eligible children.

For peer review only

Over 90% in both arms were from the most deprived areas, in keeping with the location and ethnic composition of the population. Around one in five (n=115) were overweight or obese. This proportion was slightly higher in the control (21.7%) compared to the intervention (18.3%) schools. A similar pattern was seen for other measures of body fat (skinfold measures, bioimpedance), but not for waist circumference which was similar in intervention and control groups. Under half of the children (47.9%) undertook ≥60 minutes of MVPA. Levels of physical activity (total cpm) and duration of time spent in MVPA were slightly higher among children from control, compared with intervention schools. Total dietary energy, fruit and vegetable and sugar intake were slightly higher among children in intervention, compared with control schools.

Two-years post baseline, 254 (83.3%) children in the control and 234 (86.2%) in the intervention schools were successfully followed up. There was no significant difference in baseline weight status, MVPA, diet or sex, between those followed up, and those lost to follow up (data not shown). However, SA children were less likely to be lost to follow up (n=58; 11.9%) compared with those from other ethnic groups (n=28; 34.6%).

For peer review only

Table 2: Baseline characteristics of children measured for the BEACHeS feasibility study

Characteristic/Measure*	Intervention group: N=269<br>n (%) or mean (SD)	Control group: N=305<br>n (%) or mean (SD)
Sex (n=574)		
Male	144 (53.5)	142 (49.8)
Female	125 (46.5)	153 (50.2)
Age in years (n=574)	6.53 (0.59)	6.44 (0.58)
Ethnicity (n=574)		
Bangladeshi	36 (13.4)	46 (15.1)
Indian	22 (8.2)	5 (1.6)
Pakistani	181 (67.3)	203 (66.6)
Other	30 (11.2)	51 (16.7)
Townsend score decile (n=572)		
1 (most deprived)	250 (93.3)	285 (93.8)
2	6 (2.2)	9 (3.0)
3	8 (3.0)	5 (1.6)
4-7	4 (1.5)	5 (1.6)
BMI-SDS score (n=571)	-0.03 (1.37)	0.08 (1.39)
Weight status (n=571)		
Underweight	6 (2.3)	10 (3.3)
Healthy weight	212 (79.4)	228 (75.0)
Overweight	15 (5.6)	27 (8.9)
Obese	34 (12.7)	39 (12.8)
Waist circumference in cm (n=569)	55.6 (7.7)	55.3 (6.9)
Skinfold measures in mm		
Biceps (n=563)	7.5 (3.6)	8.0 (4.0)
Triceps (n=563)	10.9 (4.1)	11.6 (4.5)
Subscapular (n=559)	7.5 (4.4)	7.9 (5.0)
Suprailiac (n=561)	7.0 (4.4)	7.4 (4.7)
Sum of 4 skinfolds (n=556)	32.5 (14.7)	34.8 (16.8)
Thigh (n=433)	14.4 (5.5)	15.7 (6.3)
Sum of upper skinfolds (n=557)	25.7 (10.9)	27.5 (12.5)
Sum of lower skinfolds (n=433)	21.0 (8.8)	22.9 (10.1)
Bioimpedance (Ω) (n=521)	692.6 (72.5)	695.1 (80.8)
Physical activity (n=535)		
Counts/min	79.9 (23.4)	83.4 (27.3)
MVPA min/24h	52.8 (28.4)	62.9 (25.0)
≥60 mins MVPA per day (n=535)	100 (40.2)	156 (54.5)
1 day school dietary intake (n=441)		
Mean energy (KJ)	2378.2 (1619.2)	1917.1 (1821.7)
Fruit and vegetables (grams)	140.6 (121.4)	105.8 (118.7)
Sugar (grams)	41.0 (60.9)	35.3 (62.9)
1 day home dietary intake (n=174)		
Mean energy (KJ)	7021.3 (3181.1)	6058.34 (3138.2)
Fruit and vegetables (grams)	329.85 (232.2)	267.95 (193.3)
Sugar (grams)	105.36 (50.2)	93.50 (36.6)
24h dietary intake (n=173)		
Total energy (KJ)	9326.6 (3083.7)	8397.3 (4035.5)
Fruit and vegetables (grams)	475.6 (261.4)	368.7 (220.2)
Sugar (grams)	154.1 (108.7)	129.7 (85.8)

*n in this column indicates how many children had useable data for each characteristic/measure

For peer review only

Estimation of effect size to inform definitive trial

Anthropometric, physical activity and dietary measures in intervention and control groups at follow up are shown in Table 3. The proportion of children who were overweight or obese had increased in all schools from baseline to follow up (from 7.3% to 9.9%, and from 12.8% to 19.1% for overweight and obese respectively). The risk of obesity was significantly lower in the intervention compared with the control group (OR 0.41; 0.19 to 0.89). The increase in BMI z-score was also significantly lower in the intervention compared with control, after adjustment [-0.15kg/m^2 (-0.27, -0.03)] (Table 4). A similar trend was seen for all other anthropometric measures, although none were statistically significant.

The ICC for the outcome "overweight/obese" compared to non-overweight, was 0.00 (95% CI (0, 0.02), whilst for BMI z-score, the ICC was 0.01 (95% CI (0, 0.04)). Therefore, taking account of clustering in the analysis would make marginal difference to the findings.

The proportion of children who undertook ≥60 minutes MVPA reduced (from 48.8% to 27.1%) at follow up, with the reduction being greater among control (30.2%) compared with intervention (23.8%) children. The differences in physical activity levels at follow up were not significant between groups (Table 4).

Total energy intake had increased slightly at follow up (7473 KJ at baseline to 8130 KJ at follow up). There were no significant differences in dietary intake between control and intervention children, although 24 hour dietary intake data were only available for 163 (33%) children at follow up, and only 61 children had dietary data at both baseline and follow up. However, school dietary intake data were more complete (93% with follow up data and 73% with both baseline and follow up data), and children in intervention schools had significantly more fruits and vegetables and lower sugar intake, compared with those in control schools (Table 4).

As the intervention was designed to be particularly relevant to SA children, we repeated the multivariate analyses including only children of SA ethnicity. The mean differences and odd ratios for the outcomes were of a similar magnitude to the main analyses (results not shown).

For peer review only

Table 3: Anthropometric, diet and physical activity measures in intervention and control groups at follow up

Measure*	Intervention group (N=234) n (%) or mean (SD)	Control group (N=254) n (%) or mean (SD)
**Anthropometric measures**		
BMI z-score (n=488)	0.13 (1.5)	0.40 (1.5)
Weight status (n=488)		
Underweight	11 (4.7)	8 (3.2)
Healthy weight	160 (68.4)	168 (66.1)
Overweight	27 (11.5)	21 (8.3)
Obese	36 (15.4)	57 (22.4)
Waist circumference (cm) (n=472)	59.4 (9.5)	60.4 (9.1)
Skinfold measures (mm)		
Biceps (n=486)	6.9(3.5)	7.7 (3.8)
Triceps (n=485)	11.2 (4.8)	11.9 (4.6)
Subscapular (n=479)	8.5 (5.3)	9.3 (5.8)
Suprailiac (n=475)	8.8 (5.9)	9.4 (5.9)
Sum of 4 skinfolds (n=471)	35.2 (18.1)	37.6 (18.4)
Thigh (n=404)	17.3 (7.5)	18.9 (8.1)
Sum of upper skinfolds (n=479)	26.5 (12.7)	28.7 (13.1)
Sum of lower skinfolds (n=402)	25.3(11.8)	27.6 (13.1)
Bioimpedance (Ω) (n=453)	692.0 (83.1)	688.3 (81.3)
**Physical Activity (n=467)**		
Counts/min	68.7 (33.4)	71.0 (22.9)
MVPA min/24h	49.1 (21.8)	51.1 (20.2)
Achieving ≥60 mins MVPA	53 (23.6)	73 (30.2)
**Dietary intake**		
School (n=454)		
Energy (KJ)	1908.7 (831.8)S	2045.1 (777.9)
Fruit and vegetables (grams)	143.1 (135.0)	93.9 (94.0)
Sugar (grams)	25.0 (15.7)	29.8 (16.7)
Home (n=163)		
Energy (KJ)	7860.5 (4366.4)	8145.4 (4004.5)
Fruit & vegetables (grams)	367.5 (316.6)	342.0 (224.9)
Sugar (grams)	113.8 (63.7)	121.0 (56.8)
24h dietary intake (n=163)		
Energy (KJ)	9527.8 (4400.3)	9820.7 (3773.1)
Fruit & vegetables (grams)	519.1 (350.2)	446.0 (238.5)
Sugar (grams)	137.2 (64.2)	150.5 (59.9)

*n in this column indicates how many children had useable data for each measure

For peer review only

Table 4: Adjusted differences in anthropometric, diet and physical activity measures between control and intervention groups

Outcome variable*	Intervention vs. Control (adjusted for baseline)	p value	Intervention vs. Control (finally adjusted)†	p value
	OR (95% CI)		*Adjusted†OR (95% CI)*	
Obese (n=486)	0.36 (0.17, 0.77)	0.01	0.41 (0.19, 0.89)	0.02
Achieving ≥60 mins MVPA (n=441)	0.82 (0.52, 1.28)	0.38	0.74 (0.45, 1.20)	0.22
	Mean difference (95%CI)		*Adjusted† mean difference (95%CI)*	
BMI z-score (n=486)	-0.15 (-0.26, -0.03)	0.02	-0.15 (-0.27, -0.03)	0.02
Waist circumference (cm) (n=482)	-0.88 (-1.87, 0.10)	0.08	-0.86 (-1.87, 0.15)	0.09
Skinfold measures (mm)				
Biceps (n=479)	-0.48 (-0.98, 0.01)	0.06	-0.44 (-0.93, 0.06)	0.08
Triceps (n=478)	-0.14 (-0.68, 0.40)	0.61	-0.10 (-0.64, 0.45)	0.71
Subscapular (n=469)	-0.46 (-0.98, 0.06)	0.09	-0.38 (-0.89, 0.14)	0.15
Suprailiac (n=468)	-0.23 (-0.84, 0.37)	0.45	-0.23 (-0.83, 0.37)	0.46
Sum of 4 skinfolds (n=461)	-1.09 (-2.85, 0.67)	0.23	-0.97 (-2.70, 0.77)	0.27
Thigh (n=324)	-0.31 (-1.39, 0.78)	0.58	-0.27 (-1.38, 0.84)	0.63
Sum of upper skinfolds (n=468)	-0.90 (-2.21, 0.42)	0.18	-0.76 (-2.05, 0.53)	0.25
Sum of lower skinfolds (n=323)	-0.36 (-1.91, 1.19)	0.65	-0.40 (-1.98, 1.18)	0.62
Bioimpedance (Ω) (n=409)	3.33 (-5.23, 11.89)	0.45	3.50 (-5.14, 12.15)	0.43
Counts/min (increments of 20) (n=441)	-0.15# (-0.34, 0.04)	0.12	-0.18# (-0.36, 0.01)	0.06
MVPA min/24h (n=441)	1.52 (-2.14, 5.17)	0.42	0.51 (-2.97, 3.99)	0.77
School				
Energy (KJ) (n=358)	-78.78 (-240.75, 83.18)	0.34	-86.02 (-250.29, 78.20)	0.30
Fruit and vegetables (grams) (n=358)	59.88 (34.56, 85.19)	<0.001	63.35 (37.53, 89.17)	<0.001
Sugar (grams) (n=358)	-3.86 (-7.27, -0.45)	0.03	-3.86 (-7.37, -0.36)	0.03
Home				
Energy (KJ)(n=61)	1322.94 (-292.75, 2938.60)	0.12	1534.73 (-117.74, 3187.20)	0.07
Fruit & vegetables (grams) (n=61)	21.61 (-81.26, 124.47)	0.68	18.98 (-89.43, 127.40)	0.73
Sugar (grams) (n=61)	6.88 (-17.04, 30.81)	0.57	9.17 (-15.16, 33.51)	0.45
24h dietary intake				
Energy (KJ) (n=61)	883.16 (-888.31, 2654.66)	0.32	1092.36 (-723.33, 2908.09)	0.23

For peer review only

Outcome variable*	Intervention vs. Control (adjusted for baseline)	p value	Intervention vs. Control (finally adjusted)†	p value
Fruit & vegetables (grams) (n=61)	89.64 (-32.51, 211.79)	0.15	86.70 (-42.92, 216.32)	0.19
Sugar (grams) (n=61)	3.16 (-22.74, 29.07)	0.81	4.23 (-22.00, 30.47)	0.75

*n indicates the number of participants included in each finally adjusted model
†Adjusted for age, sex, ethnicity and baseline values

For peer review only

Discussion

We demonstrated the feasibility of delivering a multicomponent obesity prevention intervention targeting dietary and physical activity behaviours to a socioeconomically disadvantaged, multiethnic population of primary school aged children. The feasibility study provided an opportunity to refine and modify the programme and yielded important information on acceptability and feasibility of both the intervention and measurements required for assessing outcomes in a definitive RCT.

Strengths and limitations

This is one of few studies focusing on SA populations, which comprise the largest minority ethnic group in the UK, with higher risk of obesity and its consequences. The iterative process of intervention refinement was informed by the MRC framework for complex interventions. Whilst the framework has been used for development of other interventions in NHS settings, we have demonstrated its use in the wider community setting.

The components of the intervention were influenced by stakeholder views and available resources, thus its applicability for wider populations and settings is potentially limited. However, the multifaceted intervention aimed to modify school and family environments and included elements that have been identified as promising in systematic reviews.[7,23] Furthermore the intervention components have theoretical validity for behaviour change in any population, and the incorporated techniques are transferrable. The targeting of South Asian stakeholders for intervention development is likely to have allowed us to exclude intervention components that would not be acceptable to this sub-population. Nevertheless, the developed intervention is likely to be acceptable not only in these ethnic groups, but also in the wider UK population.

Delivery of intervention, undertaken by staff outside the research team, was non-standardised. This allowed a pragmatic approach to be tested, which could be more easily rolled out. Intervention components delivered directly to the children and through school staff (physical activity component and Villa Vitality) were more likely to have high uptake than those delivered to families (leisure taster

For peer review only

sessions or walk leader training). The complexity of delivering community based interventions targeting children probably explains why most previous trials are school based.

During the trial, all children in schools allocated to the intervention arm were exposed to the intervention components. However, only about half had consent for measurements. We found no significant differences in sex and ethnicity between consented and non-consented children. Further, the distribution of weight status among children who were measured is similar to national data for this age group[24], suggesting that selection bias was unlikely.

Intervention acceptability and feasibility

A variety of intervention techniques were incorporated with variable success. Environmental restructuring (structured physical activity and play opportunities in school) was feasible and generally accepted. Demonstration of the target behaviour and prompting practice (Villa Vitality, cooking workshops, taster activity sessions and walking groups) had mixed results. Apart from Villa Vitality which was incorporated within the school setting, there was limited participation, despite enthusiasm amongst those who did take part. At a population level, these types of intervention are less feasible to deliver, unless they are incorporated within the school setting. Providing information and prompting identification of role models were feasible and acceptable and would be replicable in a larger trial. Techniques to prompt self monitoring and rewarding successful behaviour were acceptable, but had limited success in this community setting.

During the period of intervention delivery, we used a variety of methods and involved different stakeholders (school staff, parents and children), to assess the acceptability of the intervention components. We also allowed the programme to be modified and the implementation of elements to vary in the different intervention schools. This tailoring to the local school context was critical in determining the success of the intervention. For example in one school, lunchtime supervisors were trained to deliver a structured physical activity programme at lunchtime, but did not go on to deliver the programme. Following this failure of implementation, an enthusiastic teaching assistant was trained, who successfully

For peer review only

delivered the intervention. Thus, whilst standardisation of aspects of the intervention is important, some scope for tailoring to local context in terms of implementation and delivery needs to be considered.[25]

Informing a definitive RCT

The intervention was aimed at predominantly SA populations residing in inner city settings. Despite challenges, including language barriers, 80% were successfully followed up. We demonstrated the feasibility of undertaking a wide range of anthropometric measures within school and the feasibility of Actiheart monitors for assessing physical activity in free living children (approximately 90% had usable data). Assessment of dietary intake was less successful at baseline, mainly due to language barriers and difficulties for parents in completing the forms, but the feasibility study allowed us to refine the administration of the tool, so that measurement was more complete at follow up.

Although the feasibility study was not powered to examine intervention outcomes, we did find that the direction of effect for most outcomes were in favour of the intervention, supporting the need for a definitive trial. In particular, at follow up children in intervention schools had BMI z-scores on average 0.15kg/m^2 lower than children in control schools, which is in keeping with the effect size reported in a meta-analysis of childhood obesity prevention trials.[7]

The costs of the intervention were not formally examined, as this was a feasibility study and the intervention components were being modified and tested. Nevertheless the feasibility stage provided an opportunity to consider resource requirements and to modify the intervention accordingly to inform a definitive study. In order to ensure sustainability, most intervention components were adapted from existing services commissioned by the local NHS bodies at the time (including Villa Vitality, cooking courses and training of walk leaders). The resources for training teachers to deliver structured physical activity sessions are available commercially to schools, and were compiled by the research team. The signposting information for local leisure facilities and for the weekend activities was similarly compiled by the research team, summarising already available services and facilities.

For peer review only

Conclusions

We have used the MRC framework for complex interventions to develop a childhood obesity prevention intervention that can be evaluated within the context of a cluster-RCT. Although the intervention was informed by stakeholders, and evidence and guidelines from previous literature, some elements were found not to be feasible or acceptable to participants in practice. The feasibility study was an essential step in finalising the intervention programme prior to definitive evaluation. Based on the findings from this study, a definitive cluster-RCT is currently underway to assess the clinical and cost-effectiveness of the finalised intervention in primary school children (ISRCTN97000586).

For peer review only

Acknowledgements

S Passmore (Birmingham City Council) advised the study team on identification and engaging of schools and pupils, and the tailoring of interventions to be acceptable to schools.
M Howard (Heart of Birmingham PCT) facilitated the delivery of some of the intervention components through the Primary Care Trust.
E McGee (Birmingham Community Nutrition and Dietetic Service) oversaw the delivery of the cooking workshops and contributed to their evaluation.
K Westgate and S Mayle (MRC Epidemiology Unit, Cambridge) undertook cleaning, reducing and analysing the physical activity data.
C Cleghorn (University of Leeds) contributed to the development of the CADET, oversaw staff training and administration of the tool, and undertook cleaning and analysis of the dietary data.
The sponsor of the study (University of Birmingham) had no role in the study design, data collection, data analysis, data interpretation, writing of the report, or in the decision to submit the paper.

Contributors Statement

Peymané Adab: Dr. Adab conceptualized and designed the study overall, oversaw study planning, delivery and evaluation, wrote the analysis plan and drafted and revised the paper and approved the final manuscript as submitted. She is a guarantor.

Miranda Pallan: Dr. Pallan assisted in the overall delivery of the study, designed the process evaluation, undertook the data cleaning and analysis. She has approved the final manuscript as submitted, and is also a guarantor.

Janet Cade: Professor Cade designed the dietary data assessment tool, oversaw training for researchers collecting data and the analysis of the dietary data obtained. She has approved the final manuscript as submitted.

Ulf Ekelund: Dr. Ekelund advised on physical activity assessment, provided training of researchers in collecting Actiheart data and oversaw the analysis of physical activity data from Actihearts. He has approved the final manuscript as submitted.

Timothy Barrett: Professor Barrett advised on anthropomentric measurement tools used, arranged for training of research staff to undertaken measures and advised on interpretation of the anthropometric data obtained. He has approved the final manuscript as submitted.

Amanda Daley: Dr. Daley advised on physical activity components of the intervention and use of incentives to motivate children. She has approved the final manuscript as submitted.

Jonathan Deeks: Professor Deeks provided statistical support, advised on statistical analysis and on using the data obtained to inform sample size estimation for the definitive trial. He has approved the final manuscript as submitted.

Joan Duda: Professor Duda advised on the psychological measurement instruments, advised research staff on child protection issues and contributed to shaping the physical activity components of the intervention. She has approved the final manuscript as submitted.

Paramjit Gill: Dr. Gill advised on anthropometric measurements to be included, the sampling strategy and on ethnic minority health. He has approved the final manuscript as submitted.

Jayne Parry: Professor Parry advised on process evaluation and contributed to the interpretation of qualitative data obtained. She has approved the final manuscript as submitted.

For peer review only

Raj Bhopal: Professor Bhopal advised on the anthropometric measurements, definition of the target population, tailoring of the intervention to be culturally appropriate and relevant literature on ethnicity and health. He also provided important comments on the final draft and has approved the final manuscript as submitted.

Kar Keung Cheng: Professor Cheng conceived the original idea informing this study, contributed to the planning and delivery. He has approved the final manuscript as submitted.

All authors have contributed to the design of the intervention, advised on study progress and critically revised and approved the final manuscript.

Conflict of Interest Statement

All authors declare there was no support from any organisation for the submitted work; no financial relationships with any organisations that might have an interest in the submitted work in the previous three years; no other relationships or activities that could appear to have influenced the submitted work. Amanda Daley is supported by an NIHR Senior Research Fellowship award. The other authors have no disclosures relevant to this article. The views expressed in this publication are those of the authors and not necessarily those of the NHS, The National Institute for Health Research or the Department of Health. Primary Care Clinical Sciences is a member of the NIHR National School for Primary Care Research. The funding organisation did not play any role in design and conduct of the study; collection, management, analysis, and interpretation of the data; and preparation, review, or approval of the manuscript.

Data Sharing Statement

The BEACHeS study dataset is available on request from the study investigators.

Funding source

The Birmingham healthy Eating, Active lifestyle for Children Study (BEACHeS) was funded by the National Prevention Research Initiative (NPRI, http://www.npri.org.uk), Grant no. G0501292 and we are grateful to all the funding partners for their support: British Heart Foundation; Cancer Research UK; Department of Health; Diabetes UK; Economic and Social Research Council; Medical Research Council; Research and Development Office for the Northern Ireland Health and Social Services; Chief Scientist Office, Scottish Executive Health Department; Welsh Assembly Government and World Cancer Research Fund.

Clinical Trial Registration: ISRCTN51016370. The date of trial registration was delayed because this was a feasibility study. At the time of starting the study (2006), guidance on whether registration is required was ambiguous.

Licence for Publication Statement

"I Miranda Pallan, the Corresponding Author of this article contained within the original manuscript which includes any diagrams & photographs, other illustrative material, video, film or any other material howsoever submitted by the Contributor(s) at any time and related to the Contribution ("the Contribution"), have the right to grant on behalf of all authors and do grant on behalf of all authors, a licence to the BMJ Publishing Group Ltd and its licensees, to permit this Contribution (if accepted) to be published in BMJ Open and any other BMJ Group products and to exploit all subsidiary rights, as set out in the licence at: (http://group.bmj.com/products/journals/instructions-for-authors/BMJOpen_licence.pdf)

For peer review only

References

(1) Lobstein T, Baur L, Uauy R. Obesity in children and young people: a crisis in public health. Obes Rev 2004; **5**(Suppl. 1):4-85.

(2) Baker JL, Olsen LW, Sorensen TIA. Childhood Body-Mass Index and the risk of coronary heart disease in adulthood. N Engl J Med 2007; **357**:2329-37.

(3) Stamatakis E, Wardle J, Cole TJ. Childhood obesity and overweight prevalence trends in England: evidence for growing socioeconomic disparities. Int J Obes 2010; **34**:41-7.

(4) NHS Information Centre for Health and Social Care. National Child Measurement Programme: England, 2011/12 school year. The Health and Social Care Information Centre, 2012.

(5) Dinsdale H, Ridler C, Rutter H. National Child Measurement Programme: Changes in children's body mass index between 2006/7 and 2010/11. Oxford, National Obesity Observatory, 2012.

(6) Summerbell CD, Waters E, Edmunds LD, et al. Interventions for preventing obesity in children. Cochrane Database of Systematic Reviews 2005, Issue 3, John Wiley & Sons, Ltd Chichester, UK, DOI: 10 1002/14651858 CD001871 pub2.

(7) Waters E, de Silva-Sanigorski A, Hall B J, et al. Interventions for preventing obesity in children. Cochrane Database of Systematic Reviews 2011, Issue 12, John Wiley & Sons, Ltd Chichester, UK, DOI:10 1002/14651858 CD001871 pub3.

(8) McPherson K, Marsh T, Brown M. Tackling obesities: future choices - modelling future trends in obesity and the impact on health. Report No. 2. London, Government Office for Science, 2007.

(9) Wang Y, Wu Y, Wilson RF, et al. Childhood obesity prevention programs: comparative effectiveness review and meta-analysis. Comparative Effectiveness Review No. 115. Rockville, MD, Agency for Healthcare Research and Quality, 2013.

(10) National Institute for Health and Clinical Excellence. Obesity: the prevention, identification, assessment and management of overweight and obesity in adults and children. London, 2006.

(11) Campbell M, Fitzpatrick R, Haines A, et al. Framework for design and evaluation of complex interventions to improve health. BMJ 2000; **321**:694-6.

(12) Pallan M, Parry J, Cheng KK, et al. Development of a childhood obesity prevention programme with a focus on UK South Asian Communities. Prev Med 2013; **57**:948-54.

(13) Varni JW, Limbers CA, Burwinkle TM. How can young children reliably and validly self-report their health-related quality of life?: an analysis of 8,591children across age subgroups with the PedsQL 4.0 generic core scales. Health Qual Life Outcomes 2007; **5**:1.

(14) Marsh HW, Barnes J, Cairns L, et al. Self-Description Questionnaire: Age and sex effects in the structure and level of self-concept for preadolescent children . J Educ Psychol 1984; **76**:940-56.

(15) Harter S, Pike R. The pictorial scale of perceived competence and social acceptance for young children. Child Dev 1984; **55**:1969-82.

(16) Collins ME. Body figure perceptions and preferences among preadolescent children. Int J Eat Disord 1991; **10**:199-208.

For peer review only

(17) Cade JE, Frear L, Greenwood DC. Assessment of diet in young children with an emphasis on fruit and vegetable intake: using CADET – Child and Diet Evaluation Tool. Public Health Nutr 2006; **9**:501-8.

(18) Brage S, Brage N, Franks P, et al. Reliability and validity of the combined heart rate and movement sensor Actiheart. Eur J Clin Nutr 2005; **59**:561-70.

(19) Pallan M., Parry J., Adab P. Contextual influences on the development of obesity in children: a case study of UK South Asian communities. Prev Med 2012; **54**:205-11.

(20) Michie S, Ashford S, Sniehotta FF, et al. A refined taxonomy of behaviour change techniques to help people change their physical activity and healthy eating behaviours: the CALO-RE taxonomy. Psychol Health 2011; **26**:1479-98.

(21) Cole TJ, Freeman JV, Preece MA. Body mass index reference curves for the UK, 1990. Arch Dis Child 1995; **73**:25-9.

(22) Corder K, Brage S, Wareham NJ, et al. Comparison of PAEE from combined and separate HR and movement models in children. Med Sci Sports Exerc 2005; **37**:1761-7.

(23) Lavelle HV, Mackay DF, Pell JP. Systematic review and meta-analysis of school-based interventions to reduce body mass index. J Public Health 2012; **34**:360-9.

(24) National Obesity Observatory. National Child Measurement Programme: detailed analysis of the 2007/08 national dataset. London, 2009.

(25) Hawe P, Shiell A, Riley T. Complex interventions: how "out of control" can a randomised controlled trial be? BMJ 2004; **328**:1561-3.

For peer review only

Figure 1: Flow diagram of recruitment and follow up of participants in the feasibility study

For peer review only

Title: Preventing childhood obesity, phase II feasibility study focusing on South Asians: BEACHeS

Authors: Peymané Adab (MD)[1], Miranda Pallan (PhD)[1], Janet Cade (PhD)[2], Ulf Ekelund (PhD)[3], Timothy Barrett (PhD)[4], Amanda Daley (PhD)[1], Jonathan Deeks (PhD)[1], Joan Duda (PhD)[5], Paramjit Gill (DM)[1], Jayne Parry (MD)[1], Raj Bhopal (PhD)[6], KK Cheng (PhD)[1]

Affiliations:
[1]School of Health & Population Sciences (Public Health, Epidemiology and Biostatistics and Primary Care Clinical Sciences), University of Birmingham
[2]Nutritional Epidemiology Group, School of Food Science and Nutrition, University of Leeds
[3]MRC Epidemiology Unit, Institute of Metabolic Science, Cambridge & Department of Sport Medicine, Norwegian School of Sport Sciences
[4]School of Clinical and Experimental Medicine, University of Birmingham
[5]Sport & Exercise Sciences, The University of Birmingham
[6]Edinburgh Ethnicity and Health Research Group, Centre for Population Health Sciences, University of Edinburgh

Address correspondence to:
Miranda J Pallan, Public Health, Epidemiology and Biostatistics, University of Birmingham, Edgbaston, Birmingham, B15 2TT, UK.
Tel: +441214147990
Fax: +441214147878
E-mail: m.j.pallan@bham.ac.uk

Key words: Childhood obesity prevention; feasibility study; MRC framework for complex interventions

Word count:
Abstract: 273
Manuscript: 3743

For peer review only

Abstract

Objectives: To assess feasibility and acceptability of a multifaceted, culturally appropriate intervention for preventing obesity in South Asian children, and to obtain data to inform sample size for a definitive trial.

Design: Phase II feasibility study of a complex intervention.

Setting: Eight primary schools in inner city Birmingham, UK, within populations that are predominantly South Asian.

Participants: 1090 children aged 6-8 years took part in the intervention. 571 (85.9% from South Asian background) underwent baseline measures. 85.5% (n=488) were followed up 2 years later.

Interventions: The one-year intervention consisted of school and family based activities, targeting dietary and physical activity behaviours. The intervention was modified and refined throughout the period of delivery.

Main outcome measures: Acceptability and feasibility of the intervention and of measurements required to assess outcomes in a definitive trial. The difference in BMI z-score between arms was used to inform sample size calculations for a definitive trial.

Results: Some intervention components (increasing school physical activity opportunities, family cooking skills workshops, signposting of local leisure facilities and attending day event at a football club) were feasible and acceptable. Other components were acceptable, but not feasible. Promoting walking groups was neither acceptable nor feasible. At follow up, children in the intervention compared with the control group were less likely to be obese (OR 0.41; 0.19 to 0.89), and had lower adjusted BMI z-score [-0.15 (-0.27, -0.03)].

Conclusions: The feasibility study informed components for an intervention programme. The favourable direction of outcome for weight status in the intervention group supports the need for a definitive trial. A cluster randomised controlled trial is now underway to assess the clinical and cost-effectiveness of the intervention.

For peer review only

Article Summary

Strengths and limitations of the study

- We report the findings of a feasibility study of a childhood obesity prevention intervention that focuses on primary school-aged children from South Asian communities in the UK. Despite their susceptibility to the cardiometabolic consequences of obesity, little obesity prevention research has been undertaken in these communities previously.
- The early phases of the UK Medical Research Framework for complex health interventions has guided the intervention development and evaluation process undertaken in this feasibility study.
- The feasibility and acceptability of the childhood obesity prevention intervention components was variable and context dependent, however, the exploratory nature of the study enabled us to modify and refine delivery of the intervention throughout.
- Development and evaluation of the feasibility and acceptability of the intervention was undertaken in materially disadvantaged, predominantly South Asian communities, thus its transferability would be dependent on tailoring to the specific local context.
- The final intervention programme, following modification and refinement in this feasibility study, is being definitively evaluated in an ongoing cluster-randomised controlled trial.

For peer review only

Introduction

Childhood obesity is a growing problem worldwide.[1] Apart from psychological and social problems, longitudinal studies show adverse future health consequences in children as young as 7 years old who are obese.[2] In the UK, although childhood overweight prevalence has stabilised, socioeconomic disparities have widened, with increasing trend in more deprived sub-populations.[3] Data from the national childhood surveillance programmes in England show that at school entry (age 4-5 years), 9.5% of children are obese (i.e., above 95th percentile for national reference standards), but this prevalence doubles (19.2%) by the end of primary school (age 11).[4] The rate of increase among children from South Asian (SA) ethnic groups, especially girls, is greater than that for the population as a whole (increasing trend of 1.13% and 0.66% per year for Bangladeshi and Pakistani girls respectively, compared with 0.35% yearly increase in White British).[5] Thus the primary school period presents a key phase for prevention, and SA are an important target group.

However, despite numerous systematic reviews,[6,7] reports[8,9] and guidelines,[10] evidence for effective approaches to prevention is limited, particularly among minority ethnic groups. Relevant trials suggest that multifaceted school-based interventions have potential, particularly those that also include a home or community element, but the most effective combination of components is not clear.[7,9] The need for involving stakeholders, such as families, schools and local communities, in the decision making regarding potential intervention strategies has been highlighted.[6] Furthermore, for a complex intervention such as obesity prevention, which has several interconnecting components, a rigorous and iterative phased approach is required to improve study design, execution and applicability of results. The UK Medical Research Council (MRC) proposed a framework for such interventions.[11] Given the growing problem of obesity and lack of clarity on effective approaches to

For peer review only

prevention, it would be unwise to embark on another trial without thorough attention to the early phases described in the MRC framework.

The Birmingham healthy Eating and Active lifestyle for CHildren Study (BEACHeS http://www.birmingham.ac.uk/research/activity/mds/projects/HaPS/PHEB/WAVES/BEACHeS/index.aspx), used the theoretical and modelling phases of the MRC framework to develop a multifaceted childhood obesity prevention programme, targeting SA children (phase I).[12] Here we report on the feasibility study (phase II). The aim was to assess feasibility and acceptability of the intervention. In addition we wanted to obtain data to inform a definitive (phase III) cluster-randomised controlled trial (RCT).

For peer review only

Materials and Methods

The feasibility study was conducted in eight Birmingham primary schools from 2006 to 2009. Children underwent baseline measures between December 2006 and June 2007. Four schools were selected to receive the intervention (2007/8 academic year), and the remainder had no active intervention. Follow up data were collected two years after baseline.

Setting

Birmingham is UK's second city with a high minority ethnic population (34%), one fifth being from the three main SA communities (Pakistani, Bangladeshi and Indian). We obtained a list of all Local Authority-maintained primary schools in Birmingham. Of 304 schools, 52 had ≥50% of pupils from SA background (mean 75%). These, compared with the remainder, had a higher proportion of children eligible for free school meals (FSM), indicating higher deprivation. Schools were ranked in order of FSM eligibility, and those from either extreme were successively invited until 8 agreed to take part.

Participants

Pupils from years 1 and 2 (aged 5-7 years) were invited to participate. Parents of the children were approached by letter distributed through the schools, and active opt-in consent was sought for their child to participate in measurements.

Baseline and follow up measures

Age, sex and ethnicity data (from parent report at school entry) were obtained from school records on all eligible children in participating schools. Children with consent also underwent a range of anthropometric measurements, including standing height (measured to nearest 0.1cm with a Leicester Height Measure), weight (measured to nearest 0.1 kg with aTanita bioimpedance monitor), two measures of waist circumference (measured to nearest 0.5cm),

For peer review only

and skinfold thickness at five sites (biceps, triceps, subscapular, suprailiac and thigh; measured using a Holtain calliper). Children also completed interviewer administered questionnaires (not discussed in this paper, but including: quality of life (PedsQL),[13] self concept (Marsh self-description questionnaire),[14] perceived physical competence (Harter Pictorial Scale for Young Children),[15] and body image perception (adapted Collin's Pictorial Image Scale)[16]). All measures were undertaken by trained researchers using standard protocols.

Dietary intake was assessed using the Child And Dietary Evaluation Tool (CADET)[17]; a 24-hour food tick list that has been validated against a semi-weighed diary in children aged 3-7 years. A researcher completed the CADET for children during school hours, and parents were given instructions for completing it for the remainder of the 24 hour period. Physical activity levels were assessed using the Actiheart monitor (CamnTech, Papworth UK) worn for five consecutive days, including a weekend. This is validated for use in children[18] and was set up to measure acceleration and heart rate at 30 second epochs. In addition, parents were asked to complete questionnaires which included questions on family composition, and family dietary and physical activity habits.

Intervention

The process for intervention development has been reported elsewhere,[12] but in brief, the multicomponent intervention was developed by combining evidence from the literature with views from key stakeholders drawn from SA communities (including parents, teachers, school nurses, dieticians, community leaders, school governors, and retail and leisure representatives close to schools) and a multi-disciplinary group of relevant professionals. Important contextual data were gained from stakeholders, which was critical for informing intervention development and highlighted potential barriers (e.g. cultural unacceptability of certain types of physical activity for girls), as well as opportunities for intervention (e.g.

For peer review only

schools being considered a natural environment for providing skills to families), in relation to SA communities.[12,19] A review of local facilities, resources and opportunities related to healthy eating and the promotion of physical activity targeting children was used to inform the design and encourage longer term sustainability of the intervention. We also took account of national childhood obesity prevention policy during the development process to try and ensure that the intervention had an impact that was additional to existing national initiatives. The intervention targeted both diet and physical activity behaviours and consisted of two main strands: i) increasing children's physical activity levels and promoting healthy eating through schools, and ii) increasing skills among family members through family educational activities. A number of intervention techniques (as defined in the CALO-RE Taxonomy of behaviour changed techniques for physical activity and healthy eating[20]) were utilised to deliver each intervention component. A more detailed description of the intervention is provided in Table 1.

For peer review only

Table 1: Intervention components and techniques[20] included in the BEACHeS intervention programme and findings from the process evaluation

Intervention component	Aim	Intervention techniques	Description	Agent responsible for delivery	Evaluation method	Evaluation findings
			**School based activities**			
Physical activities within school day	To increase the amount of time that children are physically active within the school day	Environmental restructuring<br>Prompt practice	Three elements introduced into schools:<br>1. 'Wake Up Shake Up': a short (10 minutes) organised daily dance or exercise routine to music<br>2. Organised playground activities at lunch and break times through the training of school staff to act as "play leaders"<br>3. 'Take 10': teaching resource which links 10 minutes physical activity in the classroom to curricular subjects.	Trained school staff (including teachers, teaching assistants or lunch time assistants). The decision of which staff members to train for this component took into account individual school circumstances and was made in consultation with each school.	• Interviews with school staff<br>• Observation of sessions in schools<br>• Self-completion questionnaires administered to children and parents	Overall, school staff with a responsibility for health were enthusiastic and committed to introducing these schemes, and all schemes were acceptable to children. Individual school and staff factors strongly influence the success of each element in the different schools. Parents, in general felt that the amount of physical activity their children were undertaking in school had increased over the last year.
Incentive scheme to encourage physical activity out of school	To increase the amount of time outside of school hours that children spend doing leisure physical activities	Prompt self monitoring of behaviour<br>Prompt practice<br>Provide rewards contingent on successful behaviour	Children received a sticker collection card from school and information on local participating sports and leisure venues. Each time a child attended a venue, they collected a sticker. The child with the most stickers in each school received a prize.	Sticker collection card delivered through school class teacher. Stickers handed out by staff at leisure venues.	• Interviews with school staff<br>• Telephone survey of leisure venue staff<br>• Assessment of returned collection cards<br>• Questionnaires to children	Although this type of incentive scheme appears acceptable to children, parents and school staff alike, it was not feasible in terms of maintaining cooperation of participating venues. An element that was well received and could be retained, is the signposting information given to children and families.
Attendance at a course run by a Premier league	To encourage physical activity and healthy eating through	Provide information on consequences of behaviour<br>Model/demonstrate	School classes attend a 'Villa Vitality' day. Half the day is spent with Football Club coaches, exercising and learning football	Aston Villa Football Club Community programme staff deliver on day of visit to club.	• Interviews with school staff<br>• Self-completion questionnaires to parents	This was highly acceptable to children and school staff and is feasible to deliver to the target age group. There is some evidence that it may favourably alter children's health-

For peer review only

football club	an iconic sporting institution	behaviour<br>Prompt identification of role model/advocate<br>Goal setting (behaviour)	skills, and the other half of the day is an interactive learning session on healthy eating and healthy lifestyles. Teachers provided with material to deliver over 6 weeks to reinforce messages, and encourage weekly challenges or goals.	School class teachers deliver 6 weekly lessons after club visit.	• Pre- and 6-week post intervention questionnaires (knowledge, attitudes & behaviour) administered to children	behaviours.
**Increasing skills of families through activity-based learning**						
Cooking courses for family members	To increase healthy cooking skills and confidence and influence dietary behaviour	Provide information on consequences of behaviour in general<br>Model/demonstrate behaviour<br>Provide instruction on how to perform the behaviour<br>Prompt generalisation of behaviour	Five week courses on healthy cooking were delivered through schools to parents or other family members, some courses include children. Courses ran successively to allow all parents to attend if they wanted. Healthy recipes were distributed to support the course content.	Birmingham Community NHS Trust dietetics staff.	• Interviews with school staff<br>• Uptake rates for courses<br>• Participant pre and post-course questionnaires	This component was popular with those who participated and there was some evidence that it influenced confidence and cooking practices. Running sessions for parents and children was the most popular model, and having the sessions based in school time for children and inviting parents to attend improved attendance.
Information on local leisure opportunities and "taster" sessions for families	To equip families with the knowledge and skills to undertake physical activities with their children in their leisure time	Provide information on when and where to perform behaviour<br>Model/demonstrate behaviour	Parents were given information on local sporting and leisure venues and events. They were invited to attend weekend taster sessions with their children, through school. Activities range from cricket and football, to archery, climbing and dry-slope skiing. There was no cost for the activities and transport was provided.	BEACHeS research staff compiled list of venues and prepared signposting sheets. BEACHeS research staff accompanied families to leisure venues, where leisure venue staff delivered sessions.	• Interviews with school staff<br>• Uptake of the taster sessions<br>• Self-completion questionnaires to parents and children	This component was resource intensive to deliver, and uptake was very low. However, the signposting information was used by families, and was appreciated.

For peer review only

Training walk leaders to initiate community walking programmes	To increase walking by families and other community members through organised leisure walks	Model/demonstrate behaviour<br>Prompt practice	Community volunteers were recruited through schools to become trained walk leaders. Training was provided to equip volunteers to organise and lead walks in their local community.	Walk-leader training programme delivered through Heart of Birmingham NHS Trust.	• Assessment of numbers attending training to be walk leaders<br>• Monitoring numbers joining walking groups	This component proved unfeasible, as there was a lack of volunteers. Even those who expressed an initial interest failed to attend the training. Despite repeated efforts to recruit community volunteers, no one attended the training in any of the four BEACHeS intervention communities.

For peer review only

Allocation of intervention

This was a non-randomised feasibility trial. After baseline measurements were completed, schools were allocated to intervention or control arms. We matched schools by size, and proportion of children eligible for FSM. We then took the geographical location of the schools into account and allocated the matched pairs to either the intervention or control arm so that we minimised the chance of contamination between the two arms.

Process measures

The main aim of the study was to assess intervention feasibility and acceptability. Each component was evaluated separately, using a variety of methods. These included collection of uptake data, direct observation, questionnaires to children and parents and interviews with key school staff. The questionnaires were also used to evaluate overall perceptions of the intervention and engagement with different intervention components. Topics covered in the semi-structured interviews included exploration of how the different intervention components were implemented, which elements were perceived to work well and ideas for further development. The interviews were tape-recorded, transcribed and analysed thematically.

Other measures and analysis

We assessed the feasibility of obtaining outcome data, primarily body mass index (BMI), but also diet and physical activity and other anthropometric measures as described above. Exploratory comparison between intervention and control children was also undertaken to determine effect size.

Height and weight data were used to calculate BMI (kg/m^2) and converted into standard deviation scores (BMI z-score) using the UK 1990 growth reference charts.[21] Children were categorised as underweight, healthy weight, overweight or obese using the 2nd, 85th and 95th centile cut-offs. For waist circumference and skinfolds, the mean was used for analyses.

For peer review only

Skinfold measures were combined to obtain sum, upper (biceps, triceps and subscapular) and lower (suprailiac and thigh) skinfolds.

Data from the CADET were coded and analysed by a food diary analysis program (DANTE, University of Leeds) to estimate total energy intake (KJ) and amount of fruit and vegetables, and sugar consumed. Data on foods consumed in school and at home were analysed separately, then combined to obtain estimates for the complete 24 hour measurement period.

Accelerometry data were used to assess physical activity levels (http://www.mrc-epid.cam.ac.uk/Research/Programmes/Programme_5/InDepth/Programme%205_Downloads.html). Total daily volume of physical activity was estimated and expressed as average counts per minute (cpm). The mean duration of daily moderate to vigorous physical activity (MVPA, min/day) was calculated (400 cpm cut-off for lower threshold).[22] The proportion participating in ≥60 minutes MVPA (as recommended by international guidelines) was also calculated.

Statistical analyses were undertaken using STATA (v11). The intraclass correlation coefficient (ICC) for the main outcome (BMI z-score) was calculated, but because of the small number of schools, clustering was not taken into account for the analysis. We analysed final BMI, diet and physical activity levels of children in the intervention, compared with those in the control group. To adjust for baseline differences, we initially developed multiple linear regression models, which included the relevant baseline values of BMI, dietary factors or physical activity measures as covariates. Further models were then developed which also included potential confounders as covariates (age, sex, ethnicity). Logistic regression was used to assess risk of obesity (compared with all non-obese children), and likelihood of meeting ≥60 minutes MVPA at follow up in the intervention, compared with control children.

Ethical approval

For peer review only

Ethical approval was granted by The Black Country Research Ethics Committee (08/H1202/22). Approval was sought for active consent from parents for the child measurements, whilst consent for participation in the intervention was at school level.

For peer review only

Results

Feasibility and acceptability of intervention components

Some intervention components (particularly those delivered through school) were more successfully delivered than others. Intervention was modified during the course of delivery to optimise participation and in response to feedback. The findings are summarised in Table 1, and details are reported in the appendix.

Two intervention components were found to be unsuitable to include within an intervention programme in the format delivered. First was the scheme to incentivise out of school leisure activities. Poor co-operation from leisure venues and lack of resources to continually remind and motivate children contributed to the failure of this component. Second was the training of walk leaders. Despite effort to recruit through school staff, influential parents and various forms of publicity, volunteers were not forthcoming. The only person who underwent training did not undertake any walking groups.

One component was partially successful. Signposting of leisure facilities in the local area was popular among parents and school staff. However, attendance at organised taster sessions was poor, which was outweighed by the high staff and monetary resources required to deliver the component. The taster sessions were therefore not feasible to include in a larger trial.

The other intervention components had varying degrees of success, and the process evaluation highlighted how delivery could be improved. Individual school characteristics and differences between staff members strongly influenced the success of each element in the different schools.

For peer review only

Acceptability of allocation to control

Acceptability of non-intervention was assessed through interviews with control school staff. All understood the need for a control arm. One would have liked alternative support to compensate for not being offered the intervention. In other control schools, staff expressed that being part of the study had benefitted them in other ways, and contributed to the school's status as a 'healthy school'.

Outcomes

Feasibility of outcome measures

There were 1090 eligible children in the eight participating schools (range 54-180). Of these, 606 (55.6%) had parental consent and anthropometric measures were completed on those in school on measurement days (n=571, 94.2% of consented). Useable data (≥3 days) for Actiheart were available for 508 (89.0%). Completion of CADET was more variable. Although 445 (77.9%) were returned at baseline, 269 (47.1%) were complete, of which two thirds (n=174) had usable data. Two years after the baseline measures, 488 children (85.5%) were successfully followed up. The proportion with usable Actiheart data was similar to baseline. However a higher proportion (n=454, 93%) had a completed CADET, although only 163 (36%) had usable home data.

Findings from the feasibility study

A total of 574 children were included in the trial (Figure 1), of whom 85.9% were SA. Baseline characteristics are summarised in Table 2 (anthropometric measures were completed for 571 of the 574 participating children). The age, sex and ethnicity of those who took part were similar in distribution to the characteristics of the non-consented eligible children.

For peer review only

Over 90% in both arms were from the most deprived areas, in keeping with the location and ethnic composition of the population. Around one in five (n=115) were overweight or obese. This proportion was slightly higher in the control (21.7%) compared to the intervention (18.3%) schools. A similar pattern was seen for other measures of body fat (skinfold measures, bioimpedance), but not for waist circumference which was similar in intervention and control groups. Under half of the children (47.9%) undertook ≥60 minutes of MVPA. Levels of physical activity (total cpm) and duration of time spent in MVPA were slightly higher among children from control, compared with intervention schools. Total dietary energy, fruit and vegetable and sugar intake were slightly higher among children in intervention, compared with control schools.

Two-years post baseline, 254 (83.3%) children in the control and 234 (86.2%) in the intervention schools were successfully followed up. There was no significant difference in baseline weight status, MVPA, diet or sex, between those followed up, and those lost to follow up (data not shown). However, SA children were less likely to be lost to follow up (n=58; 11.9%) compared with those from other ethnic groups (n=28; 34.6%).

For peer review only

Table 2: Baseline characteristics of children measured for the BEACHeS feasibility study

Characteristic/Measure*	Intervention group: N=269 n (%) or mean (SD)	Control group: N=305 n (%) or mean (SD)
Sex (n=574)		
Male	144 (53.5)	142 (49.8)
Female	125 (46.5)	153 (50.2)
Age in years (n=574)	6.53 (0.59)	6.44 (0.58)
Ethnicity (n=574)		
Bangladeshi	36 (13.4)	46 (15.1)
Indian	22 (8.2)	5 (1.6)
Pakistani	181 (67.3)	203 (66.6)
Other	30 (11.2)	51 (16.7)
Townsend score decile (n=572)		
1 (most deprived)	250 (93.3)	285 (93.8)
2	6 (2.2)	9 (3.0)
3	8 (3.0)	5 (1.6)
4-7	4 (1.5)	5 (1.6)
BMI-SDS score (n=571)	-0.03 (1.37)	0.08 (1.39)
Weight status (n=571)		
Underweight	6 (2.3)	10 (3.3)
Healthy weight	212 (79.4)	228 (75.0)
Overweight	15 (5.6)	27 (8.9)
Obese	34 (12.7)	39 (12.8)
Waist circumference in cm (n=569)	55.6 (7.7)	55.3 (6.9)
Skinfold measures in mm		
Biceps (n=563)	7.5 (3.6)	8.0 (4.0)
Triceps (n=563)	10.9 (4.1)	11.6 (4.5)
Subscapular (n=559)	7.5 (4.4)	7.9 (5.0)
Suprailiac (n=561)	7.0 (4.4)	7.4 (4.7)
Sum of 4 skinfolds (n=556)	32.5 (14.7)	34.8 (16.8)
Thigh (n=433)	14.4 (5.5)	15.7 (6.3)
Sum of upper skinfolds (n=557)	25.7 (10.9)	27.5 (12.5)
Sum of lower skinfolds (n=433)	21.0 (8.8)	22.9 (10.1)
Bioimpedance (Ω) (n=521)	692.6 (72.5)	695.1 (80.8)
Physical activity (n=535)		
Counts/min	79.9 (23.4)	83.4 (27.3)
MVPA min/24h	52.8 (28.4)	62.9 (25.0)
≥60 mins MVPA per day (n=535)	100 (40.2)	156 (54.5)
1 day school dietary intake (n=441)		
Mean energy (KJ)	2378.2 (1619.2)	1917.1 (1821.7)
Fruit and vegetables (grams)	140.6 (121.4)	105.8 (118.7)
Sugar (grams)	41.0 (60.9)	35.3 (62.9)
1 day home dietary intake (n=174)		
Mean energy (KJ)	7021.3 (3181.1)	6058.34 (3138.2)
Fruit and vegetables (grams)	329.85 (232.2)	267.95 (193.3)
Sugar (grams)	105.36 (50.2)	93.50 (36.6)
24h dietary intake (n=173)		
Total energy (KJ)	9326.6 (3083.7)	8397.3 (4035.5)
Fruit and vegetables (grams)	475.6 (261.4)	368.7 (220.2)
Sugar (grams)	154.1 (108.7)	129.7 (85.8)

*n in this column indicates how many children had useable data for each characteristic/measure

For peer review only

Estimation of effect size to inform definitive trial

Anthropometric, physical activity and dietary measures in intervention and control groups at follow up are shown in Table 3. The proportion of children who were overweight or obese had increased in all schools from baseline to follow up (from 7.3% to 9.9%, and from 12.8% to 19.1% for overweight and obese respectively). The risk of obesity was significantly lower in the intervention compared with the control group (OR 0.41; 0.19 to 0.89). The increase in BMI z-score was also significantly lower in the intervention compared with control, after adjustment [-0.15kg/m^2 (-0.27, -0.03)] (Table 4). A similar trend was seen for all other anthropometric measures, although none were statistically significant.

The ICC for the outcome "overweight/obese" compared to non-overweight, was 0.00 (95% CI (0, 0.02), whilst for BMI z-score, the ICC was 0.01 (95% CI (0, 0.04)). Therefore, taking account of clustering in the analysis would make marginal difference to the findings.

The proportion of children who undertook ≥60 minutes MVPA reduced (from 48.8% to 27.1%) at follow up, with the reduction being greater among control (30.2%) compared with intervention (23.8%) children. The differences in physical activity levels at follow up were not significant between groups (Table 4).

Total energy intake had increased slightly at follow up (7473 KJ at baseline to 8130 KJ at follow up). There were no significant differences in dietary intake between control and intervention children, although 24 hour dietary intake data were only available for 163 (33%) children at follow up, and only 61 children had dietary data at both baseline and follow up. However, school dietary intake data were more complete (93% with follow up data and 73% with both baseline and follow up data), and children in intervention schools had significantly more fruits and vegetables and lower sugar intake, compared with those in control schools (Table 4).

For peer review only

As the intervention was designed to be particularly relevant to SA children, we repeated the multivariate analyses including only children of SA ethnicity. The mean differences and odd ratios for the outcomes were of a similar magnitude to the main analyses (results not shown).

For peer review only

Table 3: Anthropometric, diet and physical activity measures in intervention and control groups at follow up

Measure*	Intervention group (N=234) n (%) or mean (SD)	Control group (N=254) n (%) or mean (SD)
**Anthropometric measures**		
BMI z-score (n=488)	0.13 (1.5)	0.40 (1.5)
Weight status (n=488)		
Underweight	11 (4.7)	8 (3.2)
Healthy weight	160 (68.4)	168 (66.1)
Overweight	27 (11.5)	21 (8.3)
Obese	36 (15.4)	57 (22.4)
Waist circumference (cm) (n=472)	59.4 (9.5)	60.4 (9.1)
Skinfold measures (mm)		
Biceps (n=486)	6.9(3.5)	7.7 (3.8)
Triceps (n=485)	11.2 (4.8)	11.9 (4.6)
Subscapular (n=479)	8.5 (5.3)	9.3 (5.8)
Suprailiac (n=475)	8.8 (5.9)	9.4 (5.9)
Sum of 4 skinfolds (n=471)	35.2 (18.1)	37.6 (18.4)
Thigh (n=404)	17.3 (7.5)	18.9 (8.1)
Sum of upper skinfolds (n=479)	26.5 (12.7)	28.7 (13.1)
Sum of lower skinfolds (n=402)	25.3(11.8)	27.6 (13.1)
Bioimpedance (Ω) (n=453)	692.0 (83.1)	688.3 (81.3)
**Physical Activity (n=467)**		
Counts/min	68.7 (33.4)	71.0 (22.9)
MVPA min/24h	49.1 (21.8)	51.1 (20.2)
Achieving ≥60 mins MVPA	53 (23.6)	73 (30.2)
**Dietary intake**		
School (n=454)		
Energy (KJ)	1908.7 (831.8)S	2045.1 (777.9)
Fruit and vegetables (grams)	143.1 (135.0)	93.9 (94.0)
Sugar (grams)	25.0 (15.7)	29.8 (16.7)
Home (n=163)		
Energy (KJ)	7860.5 (4366.4)	8145.4 (4004.5)
Fruit & vegetables (grams)	367.5 (316.6)	342.0 (224.9)
Sugar (grams)	113.8 (63.7)	121.0 (56.8)
24h dietary intake (n=163)		
Energy (KJ)	9527.8 (4400.3)	9820.7 (3773.1)
Fruit & vegetables (grams)	519.1 (350.2)	446.0 (238.5)
Sugar (grams)	137.2 (64.2)	150.5 (59.9)

*n in this column indicates how many children had useable data for each measure

For peer review only

Table 4: Adjusted differences in anthropometric, diet and physical activity measures between control and intervention groups

Outcome variable*	Intervention vs. Control (adjusted for baseline)	p value	Intervention vs. Control (finally adjusted)†	p value
	OR (95% CI)		*Adjusted†OR (95% CI)*	
Obese (n=486)	0.36 (0.17, 0.77)	0.01	0.41 (0.19, 0.89)	0.02
Achieving ≥60 mins MVPA (n=441)	0.82 (0.52, 1.28)	0.38	0.74 (0.45, 1.20)	0.22
	Mean difference (95%CI)		*Adjusted† mean difference (95%CI)*	
BMI z-score (n=486)	-0.15 (-0.26, -0.03)	0.02	-0.15 (-0.27, -0.03)	0.02
Waist circumference (cm) (n=482)	-0.88 (-1.87, 0.10)	0.08	-0.86 (-1.87, 0.15)	0.09
Skinfold measures (mm)				
Biceps (n=479)	-0.48 (-0.98, 0.01)	0.06	-0.44 (-0.93, 0.06)	0.08
Triceps (n=478)	-0.14 (-0.68, 0.40)	0.61	-0.10 (-0.64, 0.45)	0.71
Subscapular (n=469)	-0.46 (-0.98, 0.06)	0.09	-0.38 (-0.89, 0.14)	0.15
Suprailiac (n=468)	-0.23 (-0.84, 0.37)	0.45	-0.23 (-0.83, 0.37)	0.46
Sum of 4 skinfolds (n=461)	-1.09 (-2.85, 0.67)	0.23	-0.97 (-2.70, 0.77)	0.27
Thigh (n=324)	-0.31 (-1.39, 0.78)	0.58	-0.27 (-1.38, 0.84)	0.63
Sum of upper skinfolds (n=468)	-0.90 (-2.21, 0.42)	0.18	-0.76 (-2.05, 0.53)	0.25
Sum of lower skinfolds (n=323)	-0.36 (-1.91, 1.19)	0.65	-0.40 (-1.98, 1.18)	0.62
Bioimpedance (Ω) (n=409)	3.33 (-5.23, 11.89)	0.45	3.50 (-5.14, 12.15)	0.43
Counts/min (increments of 20) (n=441)	-0.15# (-0.34, 0.04)	0.12	-0.18# (-0.36, 0.01)	0.06
MVPA min/24h (n=441)	1.52 (-2.14, 5.17)	0.42	0.51 (-2.97, 3.99)	0.77
School				
Energy (KJ) (n=358)	-78.78 (-240.75, 83.18)	0.34	-86.02 (-250.29, 78.20)	0.30
Fruit and vegetables (grams) (n=358)	59.88 (34.56, 85.19)	<0.001	63.35 (37.53, 89.17)	<0.001
Sugar (grams) (n=358)	-3.86 (-7.27, -0.45)	0.03	-3.86 (-7.37, -0.36)	0.03
Home				
Energy (KJ)(n=61)	1322.94 (-292.75, 2938.60)	0.12	1534.73 (-117.74, 3187.20)	0.07
Fruit & vegetables (grams) (n=61)	21.61 (-81.26, 124.47)	0.68	18.98 (-89.43, 127.40)	0.73
Sugar (grams) (n=61)	6.88 (-17.04, 30.81)	0.57	9.17 (-15.16, 33.51)	0.45
24h dietary intake				
Energy (KJ) (n=61)	883.16 (-888.31, 2654.66)	0.32	1092.36 (-723.33, 2908.09)	0.23

For peer review only

Outcome variable*	Intervention vs. Control (adjusted for baseline)	p value	Intervention vs. Control (finally adjusted)†	p value
Fruit & vegetables (grams) (n=61)	89.64 (-32.51, 211.79)	0.15	86.70 (-42.92, 216.32)	0.19
Sugar (grams) (n=61)	3.16 (-22.74, 29.07)	0.81	4.23 (-22.00, 30.47)	0.75

*n indicates the number of participants included in each finally adjusted model

†Adjusted for age, sex, ethnicity and baseline values

For peer review only

Discussion

We demonstrated the feasibility of delivering a multicomponent obesity prevention intervention targeting dietary and physical activity behaviours to a socioeconomically disadvantaged, multiethnic population of primary school aged children. The feasibility study provided an opportunity to refine and modify the programme and yielded important information on acceptability and feasibility of both the intervention and measurements required for assessing outcomes in a definitive RCT.

Strengths and limitations

This is one of few studies focusing on SA populations, which comprise the largest minority ethnic group in the UK, with higher risk of obesity and its consequences. The iterative process of intervention refinement was informed by the MRC framework for complex interventions. Whilst the framework has been used for development of other interventions in NHS settings, we have demonstrated its use in the wider community setting.

The components of the intervention were influenced by stakeholder views and available resources, thus its applicability for wider populations and settings is potentially limited. However, the multifaceted intervention aimed to modify school and family environments and included elements that have been identified as promising in systematic reviews.[7,23] Furthermore the intervention components have theoretical validity for behaviour change in any population, and the incorporated techniques are transferrable. The targeting of South Asian stakeholders for intervention development is likely to have allowed us to exclude intervention components that would not be acceptable to this sub-population. Nevertheless, the developed intervention is likely to be acceptable not only in these ethnic groups, but also in the wider UK population.

Delivery of intervention, undertaken by staff outside the research team, was non-standardised. This allowed a pragmatic approach to be tested, which could be more easily rolled out. Intervention components delivered directly to the children and through school staff (physical activity component and Villa Vitality) were more likely to have high uptake than those delivered to families (leisure taster

For peer review only

sessions or walk leader training). The complexity of delivering community based interventions targeting children probably explains why most previous trials are school based.

During the trial, all children in schools allocated to the intervention arm were exposed to the intervention components. However, only about half had consent for measurements. We found no significant differences in sex and ethnicity between consented and non-consented children. Further, the distribution of weight status among children who were measured is similar to national data for this age group[24], suggesting that selection bias was unlikely.

Intervention acceptability and feasibility

A variety of intervention techniques were incorporated with variable success. Environmental restructuring (structured physical activity and play opportunities in school) was feasible and generally accepted. Demonstration of the target behaviour and prompting practice (Villa Vitality, cooking workshops, taster activity sessions and walking groups) had mixed results. Apart from Villa Vitality which was incorporated within the school setting, there was limited participation, despite enthusiasm amongst those who did take part. At a population level, these types of intervention are less feasible to deliver, unless they are incorporated within the school setting. Providing information and prompting identification of role models were feasible and acceptable and would be replicable in a larger trial. Techniques to prompt self monitoring and rewarding successful behaviour were acceptable, but had limited success in this community setting.

During the period of intervention delivery, we used a variety of methods and involved different stakeholders (school staff, parents and children), to assess the acceptability of the intervention components. We also allowed the programme to be modified and the implementation of elements to vary in the different intervention schools. This tailoring to the local school context was critical in determining the success of the intervention. For example in one school, lunchtime supervisors were trained to deliver a structured physical activity programme at lunchtime, but did not go on to deliver the programme. Following this failure of implementation, an enthusiastic teaching assistant was trained, who successfully

For peer review only

delivered the intervention. Thus, whilst standardisation of aspects of the intervention is important, some scope for tailoring to local context in terms of implementation and delivery needs to be considered.[25]

Informing a definitive RCT

The intervention was aimed at predominantly SA populations residing in inner city settings. Despite challenges, including language barriers, 80% were successfully followed up. We demonstrated the feasibility of undertaking a wide range of anthropometric measures within school and the feasibility of Actiheart monitors for assessing physical activity in free living children (approximately 90% had usable data). Assessment of dietary intake was less successful at baseline, mainly due to language barriers and difficulties for parents in completing the forms, but the feasibility study allowed us to refine the administration of the tool, so that measurement was more complete at follow up.

Although the feasibility study was not powered to examine intervention outcomes, we did find that the direction of effect for most outcomes were in favour of the intervention, supporting the need for a definitive trial. In particular, at follow up children in intervention schools had BMI z-scores on average 0.15kg/m^2 lower than children in control schools, which is in keeping with the effect size reported in a meta-analysis of childhood obesity prevention trials.[7]

The costs of the intervention were not formally examined, as this was a feasibility study and the intervention components were being modified and tested. Nevertheless the feasibility stage provided an opportunity to consider resource requirements and to modify the intervention accordingly to inform a definitive study. In order to ensure sustainability, most intervention components were adapted from existing services commissioned by the local NHS bodies at the time (including Villa Vitality, cooking courses and training of walk leaders). The resources for training teachers to deliver structured physical activity sessions are available commercially to schools, and were compiled by the research team. The signposting information for local leisure facilities and for the weekend activities was similarly compiled by the research team, summarising already available services and facilities.

For peer review only

Conclusions

We have used the MRC framework for complex interventions to develop a childhood obesity prevention intervention that can be evaluated within the context of a cluster-RCT. Although the intervention was informed by stakeholders, and evidence and guidelines from previous literature, some elements were found not to be feasible or acceptable to participants in practice. The feasibility study was an essential step in finalising the intervention programme prior to definitive evaluation. Based on the findings from this study, a definitive cluster-RCT is currently underway to assess the clinical and cost-effectiveness of the finalised intervention in primary school children (ISRCTN97000586).

For peer review only

Acknowledgements

S Passmore (Birmingham City Council) advised the study team on identification and engaging of schools and pupils, and the tailoring of interventions to be acceptable to schools.
M Howard (Heart of Birmingham PCT) facilitated the delivery of some of the intervention components through the Primary Care Trust.
E McGee (Birmingham Community Nutrition and Dietetic Service) oversaw the delivery of the cooking workshops and contributed to their evaluation.
K Westgate and S Mayle (MRC Epidemiology Unit, Cambridge) undertook cleaning, reducing and analysing the physical activity data.
C Cleghorn (University of Leeds) contributed to the development of the CADET, oversaw staff training and administration of the tool, and undertook cleaning and analysis of the dietary data.
The sponsor of the study (University of Birmingham) had no role in the study design, data collection, data analysis, data interpretation, writing of the report, or in the decision to submit the paper.

Contributors Statement

Peymané Adab: Dr. Adab conceptualized and designed the study overall, oversaw study planning, delivery and evaluation, wrote the analysis plan and drafted and revised the paper and approved the final manuscript as submitted. She is a guarantor.

Miranda Pallan: Dr. Pallan assisted in the overall delivery of the study, designed the process evaluation, undertook the data cleaning and analysis. She has approved the final manuscript as submitted, and is also a guarantor.

Janet Cade: Professor Cade designed the dietary data assessment tool, oversaw training for researchers collecting data and the analysis of the dietary data obtained. She has approved the final manuscript as submitted.

Ulf Ekelund: Dr. Ekelund advised on physical activity assessment, provided training of researchers in collecting Actiheart data and oversaw the analysis of physical activity data from Actihearts. He has approved the final manuscript as submitted.

Timothy Barrett: Professor Barrett advised on anthropomentric measurement tools used, arranged for training of research staff to undertaken measures and advised on interpretation of the anthropometric data obtained. He has approved the final manuscript as submitted.

Amanda Daley: Dr. Daley advised on physical activity components of the intervention and use of incentives to motivate children. She has approved the final manuscript as submitted.

Jonathan Deeks: Professor Deeks provided statistical support, advised on statistical analysis and on using the data obtained to inform sample size estimation for the definitive trial. He has approved the final manuscript as submitted.

Joan Duda: Professor Duda advised on the psychological measurement instruments, advised research staff on child protection issues and contributed to shaping the physical activity components of the intervention. She has approved the final manuscript as submitted.

Paramjit Gill: Dr. Gill advised on anthropometric measurements to be included, the sampling strategy and on ethnic minority health. He has approved the final manuscript as submitted.

Jayne Parry: Professor Parry advised on process evaluation and contributed to the interpretation of qualitative data obtained. She has approved the final manuscript as submitted.

For peer review only

Raj Bhopal: Professor Bhopal advised on the anthropometric measurements, definition of the target population, tailoring of the intervention to be culturally appropriate and relevant literature on ethnicity and health. He also provided important comments on the final draft and has approved the final manuscript as submitted.

Kar Keung Cheng: Professor Cheng conceived the original idea informing this study, contributed to the planning and delivery. He has approved the final manuscript as submitted.

All authors have contributed to the design of the intervention, advised on study progress and critically revised and approved the final manuscript.

Conflict of Interest Statement

All authors declare there was no support from any organisation for the submitted work; no financial relationships with any organisations that might have an interest in the submitted work in the previous three years; no other relationships or activities that could appear to have influenced the submitted work. Amanda Daley is supported by an NIHR Senior Research Fellowship award. The other authors have no disclosures relevant to this article. The views expressed in this publication are those of the authors and not necessarily those of the NHS, The National Institute for Health Research or the Department of Health. Primary Care Clinical Sciences is a member of the NIHR National School for Primary Care Research. The funding organisation did not play any role in design and conduct of the study; collection, management, analysis, and interpretation of the data; and preparation, review, or approval of the manuscript.

Data Sharing Statement

The BEACHeS study dataset is available on request from the study investigators.

Funding source

The Birmingham healthy Eating, Active lifestyle for Children Study (BEACHeS) was funded by the National Prevention Research Initiative (NPRI, http://www.npri.org.uk), Grant no. G0501292 and we are grateful to all the funding partners for their support: British Heart Foundation; Cancer Research UK; Department of Health; Diabetes UK; Economic and Social Research Council; Medical Research Council; Research and Development Office for the Northern Ireland Health and Social Services; Chief Scientist Office, Scottish Executive Health Department; Welsh Assembly Government and World Cancer Research Fund.

Clinical Trial Registration: ISRCTN51016370. The date of trial registration was delayed because this was a feasibility study. At the time of starting the study (2006), guidance on whether registration is required was ambiguous.

Licence for Publication Statement

"I Miranda Pallan, the Corresponding Author of this article contained within the original manuscript which includes any diagrams & photographs, other illustrative material, video, film or any other material howsoever submitted by the Contributor(s) at any time and related to the Contribution ("the Contribution"), have the right to grant on behalf of all authors and do grant on behalf of all authors, a licence to the BMJ Publishing Group Ltd and its licensees, to permit this Contribution (if accepted) to be published in BMJ Open and any other BMJ Group products and to exploit all subsidiary rights, as set out in the licence at: (http://group.bmj.com/products/journals/instructions-for-authors/BMJOpen_licence.pdf)

References

(1) Lobstein T, Baur L, Uauy R. Obesity in children and young people: a crisis in public health. Obes Rev 2004; **5**(Suppl. 1):4-85.

(2) Baker JL, Olsen LW, Sorensen TIA. Childhood Body-Mass Index and the risk of coronary heart disease in adulthood. N Engl J Med 2007; **357**:2329-37.

(3) Stamatakis E, Wardle J, Cole TJ. Childhood obesity and overweight prevalence trends in England: evidence for growing socioeconomic disparities. Int J Obes 2010; **34**:41-7.

(4) NHS Information Centre for Health and Social Care. National Child Measurement Programme: England, 2011/12 school year. The Health and Social Care Information Centre, 2012.

(5) Dinsdale H, Ridler C, Rutter H. National Child Measurement Programme: Changes in children's body mass index between 2006/7 and 2010/11. Oxford, National Obesity Observatory, 2012.

(6) Summerbell CD, Waters E, Edmunds LD, et al. Interventions for preventing obesity in children. Cochrane Database of Systematic Reviews 2005, Issue 3, John Wiley & Sons, Ltd Chichester, UK, DOI: 10 1002/14651858 CD001871 pub2.

(7) Waters E, de Silva-Sanigorski A, Hall B J, et al. Interventions for preventing obesity in children. Cochrane Database of Systematic Reviews 2011, Issue 12, John Wiley & Sons, Ltd Chichester, UK, DOI:10 1002/14651858 CD001871 pub3.

(8) McPherson K, Marsh T, Brown M. Tackling obesities: future choices - modelling future trends in obesity and the impact on health. Report No. 2. London, Government Office for Science, 2007.

(9) Wang Y, Wu Y, Wilson RF, et al. Childhood obesity prevention programs: comparative effectiveness review and meta-analysis. Comparative Effectiveness Review No. 115. Rockville, MD, Agency for Healthcare Research and Quality, 2013.

(10) National Institute for Health and Clinical Excellence. Obesity: the prevention, identification, assessment and management of overweight and obesity in adults and children. London, 2006.

(11) Campbell M, Fitzpatrick R, Haines A, et al. Framework for design and evaluation of complex interventions to improve health. BMJ 2000; **321**:694-6.

(12) Pallan M, Parry J, Cheng KK, Adab P. Development of a childhood obesity prevention programme with a focus on UK South Asian Communities. Prev Med 2013; **57**:948-54.

(13) Varni JW, Limbers CA, Burwinkle TM. How can young children reliably and validly self-report their health-related quality of life?: an analysis of 8,591children across age subgroups with the PedsQL 4.0 generic core scales. Health Qual Life Outcomes 2007; **5**:1.

(14) Marsh HW, Barnes J, Cairns L, Tidman M. Self-Description Questionnaire: Age and sex effects in the structure and level of self-concept for preadolescent children . J Educ Psychol 1984; **76**:940-56.

(15) Harter S, Pike R. The pictorial scale of perceived competence and social acceptance for young children. Child Dev 1984; **55**:1969-82.

For peer review only

(16) Collins ME. Body figure perceptions and preferences among preadolescent children. Int J Eat Disord 1991; **10**:199-208.

(17) Cade JE, Frear L, Greenwood DC. Assessment of diet in young children with an emphasis on fruit and vegetable intake: using CADET – Child and Diet Evaluation Tool. Public Health Nutr 2006; **9**:501-8.

(18) Brage S, Brage N, Franks P, et al. Reliability and validity of the combined heart rate and movement sensor Actiheart. Eur J Clin Nutr 2005; **59**:561-70.

(19) Pallan M., Parry J., Adab P. Contextual influences on the development of obesity in children: a case study of UK South Asian communities. Prev Med 2012; **54**:205-11.

(20) Michie S, Ashford S, Sniehotta FF, et al. A refined taxonomy of behaviour change techniques to help people change their physical activity and healthy eating behaviours: the CALO-RE taxonomy. Psychol Health 2011; **26**:1479-98.

(21) Cole TJ, Freeman JV, Preece MA. Body mass index reference curves for the UK, 1990. Arch Dis Child 1995; **73**:25-9.

(22) Corder K, Brage S, Wareham NJ, et al. Comparison of PAEE from combined and separate HR and movement models in children. Med Sci Sports Exerc 2005; **37**:1761-7.

(23) Lavelle HV, Mackay DF, Pell JP. Systematic review and meta-analysis of school-based interventions to reduce body mass index. J Public Health 2012; **34**:360-9.

(24) National Obesity Observatory. National Child Measurement Programme: detailed analysis of the 2007/08 national dataset. London, 2009.

(25) Hawe P, Shiell A, Riley T. Complex interventions: how "out of control" can a randomised controlled trial be? BMJ 2004; **328**:1561-3.

For peer review only

Figure 1: Flow diagram of recruitment and follow up of participants in the feasibility study

For peer review only

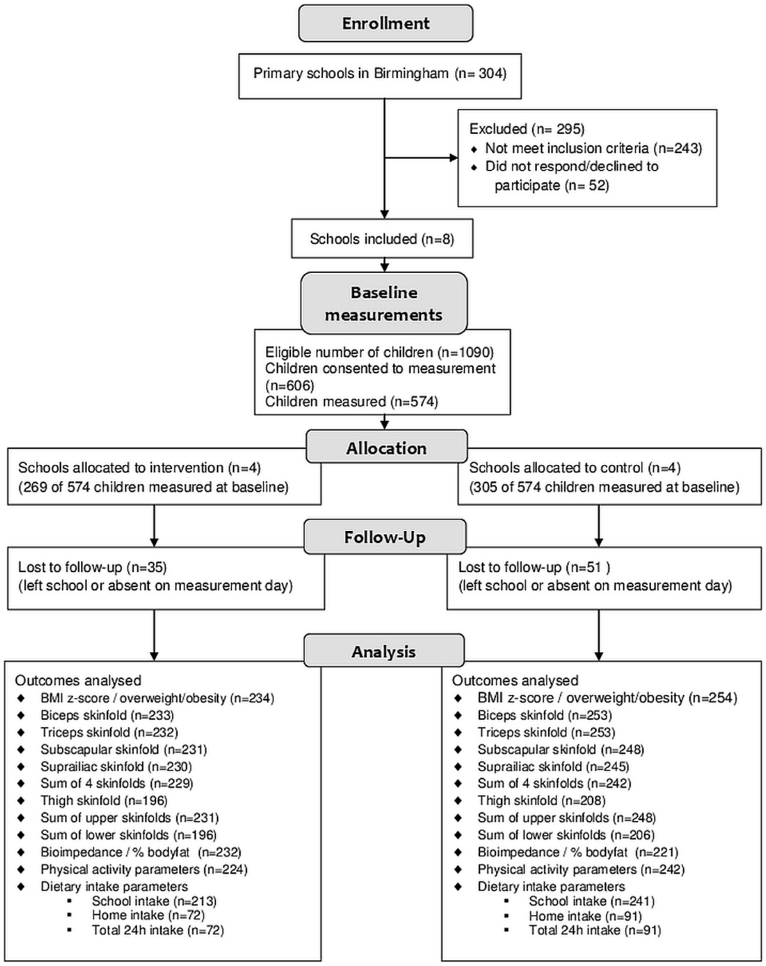

90x127mm (300 x 300 DPI)

Preventing childhood obesity, exploratory trial focusing on South Asians: BEACHeS

Appendix: Detailed findings from the process evaluation of intervention components included in the BEACHeS programme

Intervention component		Evaluation findings
**School based activities**	Physical activities within school day	***"Wake Up Shake Up":*** 3 intervention schools implemented this to varying degrees (twice a day in one school, once or less in others).<br>Participation by children was greater if session was compulsory rather than optional.<br>Questionnaire responses from the children suggested that it was generally well received, although a few children were reluctant to join in.<br>Staff were generally enthusiastic about running this, but identified lack of time and lack of space as potential barriers to success.<br>*"[Wake Up Shake Up is] really popular, going really well, parents are now starting to join in a lot more because the idea was that the parents would join in as well. So I am really pleased with how that's coming on"* (teacher and school physical education lead)<br>***Organised playground activities:*** All 4 schools received training and attempted to introduce this at lunch times. Implementation varied greatly between schools, with some schools not consistently being able to deliver.<br>A key factor for successful implementation was having an enthusiastic member of staff to inspire the play leaders. The school that had the least success with implementation was where the lunchtime supervisors were trained as play leaders but did not have a member of staff to lead them.<br>Provision of playground equipment and using a zoning system in the playground supported successful delivery.<br>Children and staff indicated that they enjoyed the scheme and supported it.<br>*"What I have found with [a teaching assistant] going out, who's doing like the parachute games and that with them is much better, because the dinner ladies just didn't take it on board. I think they came to the training because they had to but it's had no impact, I've got to be honest"* (deputy head teacher)<br>***Take 10 curricular materials:*** 2 of the schools implemented this component.<br>Success depended on the enthusiasm and motivation of the class teachers. Teachers who used Take 10 were keen to continue and expand its use.<br>Some members of staff identified lack of training and familiarity with the material as barriers to its implementation.<br>*"I know it [Take 10] works really well, I know it's really accessible....the only problems I have is with the staff doing it or not doing it"* (teacher and school physical education lead)
	Incentive scheme to encourage physical activity out of school	The major obstacle to the success of this component was retention of leisure venues and maintenance of enthusiasm.<br>Most venues that were initially recruited no longer actively participated after 2-3 months. Staff turnover, change in management, high frequency of temporary staff, low perceived importance and lack of interest from leisure centre staff contributed to the failure of this component.<br>School staff felt that incentive schemes were generally a good way of motivating children. However, in addition to problems with venue participation, other problems were identified. Teachers felt that motivation needed regular reinforcement (e.g. by class teachers), children

For peer review only

		needed a specific goal to work towards, and a shorter time frame (e.g. one term rather than one school year) and tangible reward (named item, rather than an unspecified prize) would have helped to focus the children.<br>Children were generally enthusiastic about collecting stickers. However difficulties experienced with receiving stickers from the majority of venues de-motivated them.<br>Parents had found information on local sporting and leisure venues useful. The majority of parents were also in favour of incentive schemes to encourage children to undertake physical activities.<br>*“they [the children] were so eager to collect the stickers... especially at the beginning when we were having trouble with the stickers, when they [the leisure venues] weren’t giving them”* (deputy head teacher)
	Attendance at a course run by a Premier league football club	This component was extremely popular both with children and school staff, with high (near 100%) attendance<br>Most parents did not recall that their children attended the ‘Villa Vitality’ day<br>Children attending Villa Vitality reported significantly greater levels of physical activity outside of school time 6-weeks post attendance, compared to control children.<br>*“they really enjoyed that [Villa Vitality], I think that was... you know, once they got into it and realised what it was about and that they got a lot out of it, then they really enjoyed it”* (deputy head teacher)
**Increasing skills of families through activity-based learning**	Cooking courses for family members	Uptake was generally lower than capacity (4-10 participants / course). Uptake was highest in one school with an enthusiastic parent link worker.<br>There was some drop out over the 5 weeks of the course but over 90% of participants attended 3 or more sessions.<br>The courses were well received by those who attended. Compared to parents who did not participate, participants had lower baseline confidence in their cooking ability. Following the course, participants had higher levels of reported confidence than at baseline in shopping for healthy food and cooking healthy meals. They also reported that the family was eating more healthily.<br>Courses where the children were also invited to cook with their parents were generally better attended. The model of parents learning about healthy cooking with their children was popular with parents, children and school staff.<br>School staff thought it was feasible to use school time for healthy cooking sessions for children where parents could be invited along.<br>Supporting information such as healthy eating tips and recipes in newsletters was well received by parents.<br>*“[cooking courses were] very, very successful, wonderful way of doing it. So the parents were learning about healthy ways of cooking and so on without it looking as if they were having a finger wagged at them”* (teacher and school-community liaison manager)
	Information on local leisure opportunities and week-end “taster” sessions for families	Uptake of the taster activity sessions was generally poor, although it did increase through the year through recommendations by those who attended.<br>Activities were very well received by those who did attend.<br>School staff were supportive by the taster activity sessions and expressed surprise and disappointment by the low uptake rates.<br>The taster sessions were extremely resource intensive to run, in terms of cost and staff time.<br>Most parents (>90%) found the information signposting healthy activities and venues useful, and school staff also felt this had been useful for

For peer review only

		parents and served to motivate them to try out local facilities. *"I was so disappointed that out of 120 children, so few families took up these free visits when they were actually picked up and taken somewhere"* (deputy head teacher)
	Training walk leaders to initiate community walking programmes	This component proved unfeasible, as there was a lack of volunteers to train as walk leaders. Even those who expressed an initial interest failed to attend the training. Despite repeated efforts to recruit community volunteers, only one person attended a training session, but they failed to organise any walking trips. *"we put signs up and sent leaflets out to see if anybody was interested in being a walk leader or training and I don't think we got anybody back at all"* (deputy head teacher)

For peer review only
